# Hinge Truncation to Improve Aggregation Kinetics and Thermal Stability of an Antibody Fab Fragment

**DOI:** 10.1021/acs.molpharmaceut.5c00358

**Published:** 2025-08-07

**Authors:** Cheng Zhang, Kersti Karu, Paul A. Dalby

**Affiliations:** † Department of Biochemical Engineering, 4919 University College London, Gower Street, London WC1E 6BT, U.K.; ‡ Department of Chemistry, University College London, 20 Gordon Street, London WC1H 0AJ, U.K.

**Keywords:** hinge, truncation, stability, aggregation, engineering, therapeutic

## Abstract

The hinge region of antibody fragments plays a crucial role in their stability and aggregation properties. In this study, we investigated the effects of hinge truncations on the thermal stability and aggregation propensity of the A33 Fab antibody fragment. Eight Fab variants were engineered by introducing stop codons to truncate 1–8 residues at the hinge region (heavy chain residues 221–228). These variants were then expressed, purified, and characterized in terms of stability and aggregation propensity using SDS-PAGE, SEC-HPLC, LC–MS, and thermal stability assays. Our findings demonstrate that truncating the hinge region can enhance the thermal stability and reduce the aggregation of Fab fragments, and that progressive truncations identified an optimal hinge length for stability. Notably, the 227TGA variant exhibited a significant 14.5% reduction in aggregation rate compared to the wild type, without compromising thermal stability. By contrast, 221TGA removed all of the hinge and reduced the aggregation rate by 13%, but also decreased the thermal stability. These results suggest that hinge truncation is a promising strategy for improving the developability of therapeutic antibody Fab fragments by mitigating some of the stability issues associated with aggregation.

## Graphical abstract

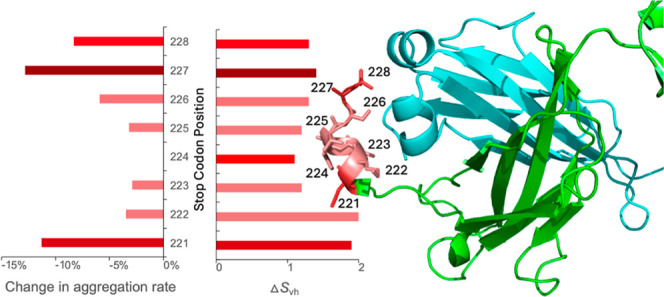

## Introduction

Antibodies are integral to a wide array of therapeutic and diagnostic applications, necessitating a focus on their stability as a critical aspect of biochemical engineering.^1–3^ The challenges of aggregation and thermal degradation continue to impact their therapeutic efficacy and shelf life, despite substantial research efforts.^4,5^ This has led to various engineering strategies aimed at enhancing their physical stability, including genetic mutations,^6,7^ the introduction of stabilizing disulfide bonds,^8,9^ and modifications to glycosylation patterns,^10,11^ which aim to improve the molecule’s robustness while balancing the potential impacts on its binding affinity and overall function.

One common method involves the strategic mutation of amino acids within the antibody structure, often targeting the complementarity-determining regions (CDR)^12–14^ or other strategic sites to decrease aggregation propensity without negatively affecting antigen-binding affinity.^15,16^ While beneficial, these mutations require careful design to avoid detrimental impacts on antibody functionality. Additionally, enhancing the structural integrity of antibodies through additional disulfide bonds aims to stabilize the protein conformation, thus reducing the likelihood of unfolding and subsequent aggregation under physiological conditions.^17^ However, this can sometimes alter the molecular structure in ways that impair binding or modify its immunogenic profile.^18^ Beyond these methods, other engineering techniques include altering the glycosylation pattern of antibodies to improve stability and reduce aggregation.^19^ While effective, these approaches often require complex biotechnological manipulations and may not be universally applicable across all antibody types.

Hinge deletion for a full antibody has been shown previously to reduce effector functions but also slightly decrease stability,^20^ yet the impact of hinge truncation in Fab fragments remains underexplored. While hinge flexibility is crucial for enabling domain movement and optimal orientation between Fab and Fc for effective antigen binding and effector function in full antibodies, it is not required in the Fab format.

Fabs are frequently utilized independently in therapeutic settings due to their smaller size and unique binding properties.^21^ The A33 antibody Fab fragment, which has been extensively studied, characterized and structurally resolved in prior research,^22–24^ serves as an ideal model protein in this investigation due to its well-documented structural properties and the known flexibility of its hinge region. It consists of a light chain (LC) and a heavy chain (HC) as shown in Figure 1. The LC contains 214 residues (LC 1–214), and the HC contains 228 residues (HC 1–228), with its C-terminus linked to the hinge region of a full IgG antibody. Recent studies have highlighted the flexibility of the HC C-terminus (referred as “hinge region” here for brevity),^25^ and have shown that mutations in this area can decrease aggregation by up to 12% compared to the wild type.^26^ These stabilizing mutations were associated with a significant increase in van’t Hoff entropy change (Δ*S*_vh_), suggesting reduced conformational flexibility and entropy in the native ensemble. Consequently, we hypothesized that an alternative strategy to rigidify the hinge region and reduce aggregation could be to partially truncate it, which not only shortens the HC C-terminus but also potentially minimizes disruption to adjacent residues. Since the hinge region is remote from the Fab CDR binding domain, careful truncation is unlikely to compromise the molecular integrity or binding affinity. In this study, we truncated 1–8 residues at the 8-residue HC hinge region (residues 221–228, or 217–224 Kabat) using a “TGA” stop codon, creating eight Fab variants with progressively shortened hinges. It is important to note that an interchain disulfide bond exists between LC214 and HC220. The 221TGA variant could potentially expose this disulfide bond to the solvent, increasing the likelihood of incorrect cysteine pairing and resulting in Fab–Fab dimer formation.

**Figure 1:**
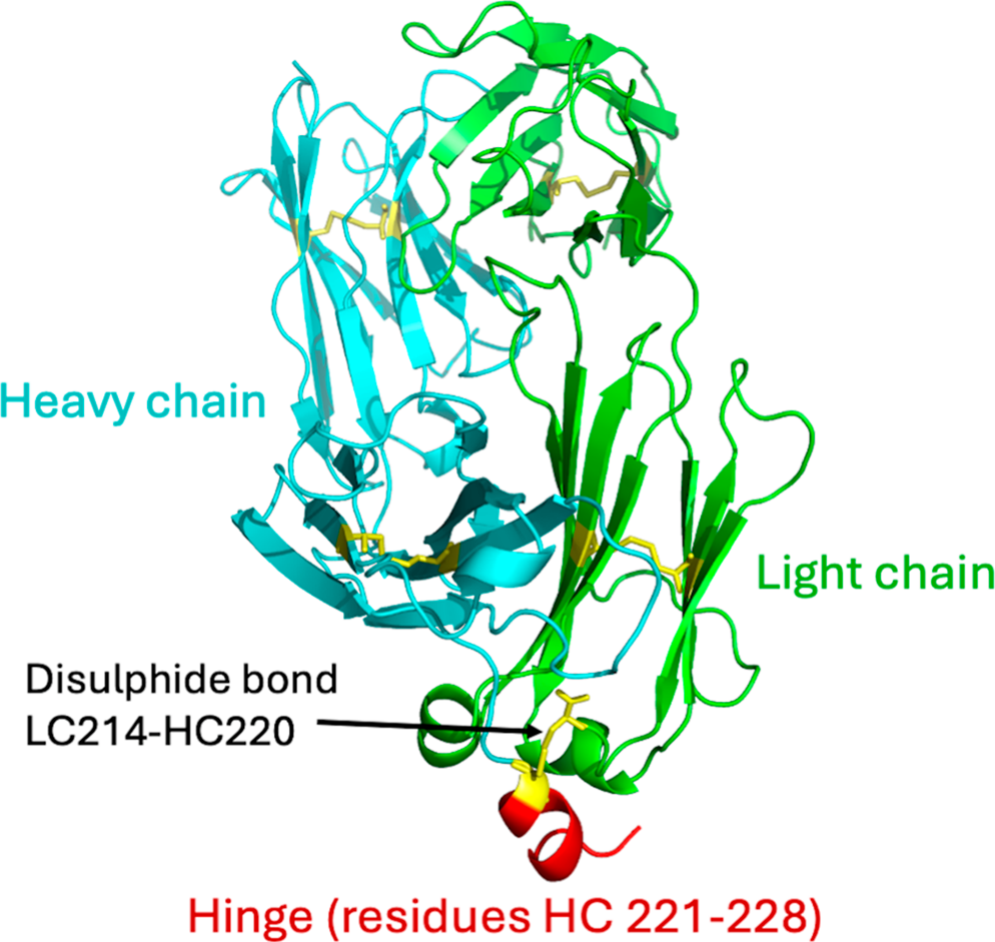
Structure of A33 Fab with modifications at the hinge region. Light chain (green) and heavy chain (cyan) are depicted in cartoon format. The five disulfide bonds are represented in sticks and colored yellow, with the interchain disulfide bond between LC214 and HC220 indicated by an arrow. Residues 221–228 of the heavy chain, highlighted in red and also indicated by an arrow, are progressively truncated from 1 to 8 residues to create eight variant structures with a shortened hinge region.

## Materials and Methods

### In Vitro Site-Directed Mutagenesis

The in vitro site-directed mutagenesis and transformation was carried out as previously reported.^26^ The QuikChange II XL Site-Directed Mutagenesis Kit (Agilent Technologies, USA) was employed for site-directed mutagenesis, after which the modified plasmids were introduced into One Shot TOP10 chemically competent *E. coli* (Thermo Fisher Scientific, UK) and cultivated on Tet + LB agar plates. Individual colonies were selected to extract and sequence plasmids. After confirming the plasmid sequences, they were reintroduced into W3110 *E. coli* strains, and glycerol stocks were created for future fermentation processes.

Stop codon TGA was used to truncate 1–8 residues at the hinge region of heavy chain, resulting in eight Fab variants with reduced lengths, and were named 228TGA, 227TGA, 226TGA, 225TGA, 224TGA, 223TGA, 222TGA, 221TGA, respectively. The mutations were verified by DNA sequencing from Source BioScience (Nottingham, UK). The amino acids and DNA sequences for the wild type Fab are shown in the Supporting Information, with the mutational positions highlighted.

### Fab Expression, Purification, and Buffer Exchange

The procedures for Fab expression from the pTTOD plasmid in *E. coli* strain W3110, with SM6G defined media, 112 g/L glycerol and 10 μg/mL tetracycline at 30 °C, and purification followed established methods,^26^ utilizing 4 × 250 mL DASbox Mini Bioreactor (Hamburg, Germany) and a Protein G chromatography column on an ÄKTA Explorer System (GE Healthcare, Buckinghamshire, UK). Tight control with established protocols for this production train ensured consistency with previous studies of the same Fab. Formulations of Fab were prepared and stabilized at 4 °C a day before conducting aggregation kinetics and thermal stability tests. Samples of Fab, previously frozen at −80 °C, were thawed and passed through Anotop 25 0.02 μm syringe filters (GE Healthcare, Buckinghamshire, UK) to eliminate aggregates. The samples were then subjected to buffer exchange with Milli-Q water, concentrated to 2 mg/mL using 30 kDa cutoff Vivaspins (Generon Ltd., Berkshire, UK), and maintained at 4 °C for storage.

### Sodium Dodecyl Sulfate-Polyacrylamide Gel Electrophoresis

SDS-PAGE was performed to analyze the Fab protein samples under both nonreducing and reducing conditions. The samples were prepared at a concentration of 2 mg/mL in water. For nonreducing conditions, 10 μL of the Fab solution was mixed with 5 μL of 4× loading buffer and 5 μL of water; for reducing conditions, 10 μL of the Fab solution was combined with 5 μL of 4× loading buffer and 5 μL of 0.2 M dithiothreitol (DTT). Both sample types were subsequently heated at 90–95 °C for 15 min and then centrifuged briefly to collect the insoluble material at the bottom of the tube.

The prepared samples were loaded onto a 4–12% polyacrylamide gradient Bis-Tris gel (NuPAGE, NP0322BOX) using a 12-well configuration. The gradient gel ensures good resolution of species from potential fragments through to aggregate molecular weights. Each well received 10 μL of the sample. For molecular weight estimation, a prestained protein ladder (PageRuler Plus, 26,619) was used, with 5 μL loaded into the leftmost well and 10 μL into the rightmost well of the gel. Electrophoresis was conducted in a NuPAGE MES SDS Running Buffer (NP0002) at 160 V for approximately 40–45 min until the desired separation was achieved.

Post-electrophoresis, the gels were stained for 1 h using SimplyBlue SafeStain (LC6060) to visualize the proteins. The gels were subsequently destained overnight to remove excess stain and enhance the visibility of the protein bands. Following destaining, the gels were examined under a UV imager (Amersham Imager 600) to assess the protein separation.

### Liquid Chromatography Mass Spectrometry

Fab protein samples were prepared at 0.2 mg/mL in water for LC–MS. An Agilent 6510 QTOF LC–MS instrument was utilized for analyses. A 10 μL sample was injected through a 100 μL loop onto a reverse phase RPLC-S, 1000 A, 8 μM, 150 × 2.1 mm column (Part No PL1912-3802) maintained at 60 °C. The sample tray temperature was kept at 4 °C. The mobile phases were 0.1% formic acid in water (A) and 100% acetonitrile in 0.1% formic acid water (B). The flow rate was 300 μL/min with gradient as follows:- 15% B for the first 2 min followed by increase to 32% B over 1 min, remained at 32% B for 1 min. After 4 min of the LC gradient, mobile phase B was increased to 50% over 10 min, with further increase of B to 95% over 4 min and maintained at 95% B for 2 min. At 22 min of the LC gradient, the mobile phase B was changed to 15% B for the next 3 min to condition the LC column for the next injection. The total LC run was 25 min. The LC effluent was directed through capillary tubing into the Agilent 6510 QTOF mass spectrometer with a capillary dual-nebulizer electrospray ion (ESI) source operating in positive-ion mode, the parameters were set as follows: Vcapillary voltage 4000 V, fragmentor voltage 215 V, skimmer voltage 65 V, oct RF Vpp 750 V, oct1 DC 45, lens1 43.9 V and lens2 32.7 V, amu 500. The ESI source parameters were nebulizer 20 psi, drying gas temp 325 °C, drying gas flow 5 L/min. The drying gas is nitrogen. The acquisition rate was 1 spectra/sec and acquisition time 1000 ms/spectrum corresponding to 9652 transients/spectrum. Data acquisition was performed in profile mode with the mass range from *m*/*z* 800 to 5000. The data was processed using maximum entropy deconvolution algorithm incorporated in MassHunter software (version B.07.00).

### Thermal Stability

Thermal stability assessments were performed using the UNcle (Unchained Laboratories, UK). Fab samples were prepared in triplicate at a concentration of 1 mg/mL in a pH 4 solution of 20 mM sodium citrate, 155 mM NaCl (for a total ionic strength of 200 mM based on theoretical calculations excluding the Fab). Each sample involved adding 20 μL of 2 mg/mL Fab dissolved in water to 20 μL of double-concentrated stock buffer. Subsequently, 9 μL from each sample was transferred into a cuvette and loaded into the UNit for analysis. The samples underwent gradual heating from 20 to 95 °C, increasing at a linear rate of 1 °C/min, and monitored by intrinsic fluorescence and static light scattering (SLS) at 266 and 473 nm. *T*_agg_ measurements were analyzed using the Uncle software.

The thermal stability parameters including the van’t Hoff enthalpy change (Δ*H*_vh_), entropy change (Δ*S*_vh_), and the midpoint of thermal unfolding (*T*_m_)—where 50% of the protein unfolds—were determined using the barycentric mean (BCM) of the intrinsic fluorescence spectra of the protein across 280–460 nm wavelengths at ramping temperature, after fitting to the two-state van’t Hoff eq 1

1 $$I_{T}=\frac{\left(I_{N}+aT\right)+\left(I_{D}+bT\right)\exp\left[\frac{\Delta H_{vh}}{R}\left(\frac{1}{T_{m}}-\frac{1}{T}\right)\right]}{1+\exp\left[\frac{\Delta H_{vh}}{R}\left(\frac{1}{T_{m}}-\frac{1}{T}\right)\right]}$$

Three-state transition eq 2 was also used to fit the thermal curves from 221TGA and 222TGA as a third transition was visible only for these vairants.

2 $$I_{T}=\frac{\left(I_{N}+aT\right)+\left(I_{I}+bT\right)\exp\left[\frac{\Delta H_{vh1}}{R}\left(\frac{1}{T_{m1}}-\frac{1}{T}\right)\right]+\left(I_{D}+cT\right)\exp\left[\frac{\Delta H_{vh1}}{R}\left(\frac{1}{T_{m1}}-\frac{1}{T}\right)\right]\exp\left[\frac{\Delta H_{vh2}}{R}\left(\frac{1}{T_{m2}}-\frac{1}{T}\right)\right]}{1+\exp\left[\frac{\Delta H_{vh1}}{R}\left(\frac{1}{T_{m1}}-\frac{1}{T}\right)\right]+\exp\left[\frac{\Delta H_{vh1}}{R}\left(\frac{1}{T_{m1}}-\frac{1}{T}\right)\right]\exp\left[\frac{\Delta H_{vh2}}{R}\left(\frac{1}{T_{m2}}-\frac{1}{T}\right)\right]}$$

where *I*_N_ is the signal at temperature *T*, *I*_N_, *I*_D_, and *I*_I_ are the signals of the native, denatured and intermediate states, *a*, *b* are the native and denatured region baseline slopes, and *R* is the molar gas constant.

### Aggregation Kinetics

A double-concentrated stock buffer was prepared as described for the thermal stability assessments and combined with 2 mg/mL of Fab in Milli-Q water to attain a final concentration of 1 mg/mL in 20 mM sodium citrate pH 4, 155 mM NaCl. Each sample, measuring 20 μL, was dispensed into a 0.2 mL thin-walled RNase-free PCR tube (Fisher Scientific, UK) and placed in a thermal cycler (Bio-Rad C1000 Touch, UK) preset at 4 °C. Isothermal program of 65 °C was initiated and samples were taken in triplicate for each variant at intervals of 5 min, immediately cooled on ice for 5 min, and then centrifuged at 21,300*g* at 4 °C for 15 min. From the resultant supernatant, 15 μL was transferred to an HPLC vial insert and 5 μL was injected into an Agilent Diol Guard Column (4.6 mm ID × 12.5 mm), followed by an Agilent Zorbax Bio Series GF-250 SEC-HPLC column (Agilent, Berkshire, UK). The SEC-HPLC operated at a flow rate of 1 mL/min using 200 mM sodium phosphate buffer at pH 7 on an Agilent 1200 HPLC system (Cheshire, UK), running each cycle for 4.5 min. Calibration curves were established with injections of 0, 1, 2, and 5 μL from a 2 mg/mL solution prior to each batch of analysis.

To model the kinetics of monomer loss, an exponential function (eq 3) was used where *A* and *k* are the coefficients, *y* represents the normalized monomer retention from 0 to 1, and *t* denotes the incubation time. The initial rate of aggregation *v* at *t* = 0 is given by the first derivative of eq 3, which is *A* × *k* in min^–1^.

3 $$y=A\times e^{\left(-kt\right)}$$

## Results and Discussion

### Assessment of Monomeric and Dimeric States of Fab Variants by SDS-PAGE, SEC-HPLC and Mass Spectrometry

Having successfully introduced the stop codons in the Fab variants, as confirmed by sequencing, it was imperative to assess the hinge truncation and overall integrity of the expressed and purified Fab variants prior to performing any forced degradation studies of aggregation. These evaluations were necessary due to the potential instability and formation of atypical oligomers in the new variants. As depicted in Figure 2, SDS-PAGE analysis was performed under both non-reducing and reducing conditions to evaluate the molecular integrity and homogeneity of the protein variants.

**Figure 2:**
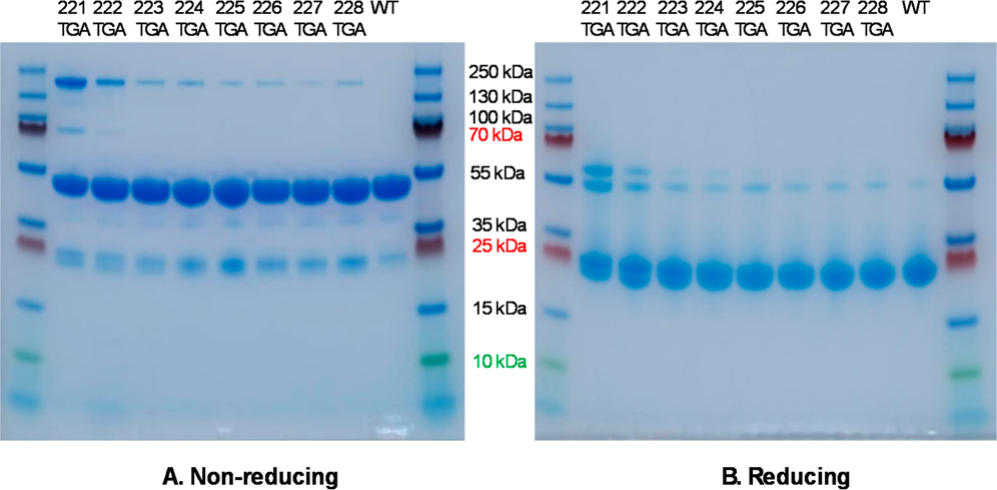
Comparative SDS-PAGE analysis under nonreducing (A) and reducing (B) conditions. Protein ladders are loaded in the outermost lanes of both gels. Eight Fab variants of 221TGA to 228TGA along with the wild type samples are loaded in the center wells, progressing from left to right.

Under non-reducing conditions, the predominant bands for all samples were observed just below 55 kDa, accompanied by minor bands just below 25 kDa. These bands corresponded to the full Fab fragments (approximately 47 kDa) and the light chain/heavy chain (LC/HC) fragments (approximately 23–24 kDa), respectively. The molecular weight differences resulting from the progressive truncations of 1–8 residues between the variants were not surprisingly indiscernible due to the resolution limitations of SDS-PAGE.

All variants showed a band at between 130 and 250 kDa, indicative of higher molecular weight species. This band was most intense for the 221TGA variant and less so for 222TGA, but then much fainter for the remaining variants 223TGA to 228TGA, and absent for wild type. The presence of this band is well beyond the expected molecular weight of a Fab–Fab dimer (93 kDa), suggesting the formation of a higher order Fab oligomer, such as a trimer (140 kDa) or tetramer (186 kDa). A weak band at around 70 kDa was exclusively observed in the 221TGA variant, suggesting unique oligomerization patterns possibly due to mis-paired cysteine residues leading to dimerization between Fab fragments and LC/HC, forming Fab-LC (70.0 kDa) and Fab-HC (69.7 kDa) species in the 221TGA variant, or possibly due to the formation of a disulfide bond with a host cell protein.

Under reducing conditions, the major band was consistently observed at slightly below 25 kDa across all variants, indicating the presence of the LC and HC fragments. For the 221TGA variant, two additional bands in the range of 55–60 kDa were detected, with a reduced intensity observed in the 222TGA variant, and progressively fainter in variants 223TGA to 228TGA and wild type. The origin of these bands remains unclear but may be attributed to proteolytic cleavage of Fab-LC or Fab-HC, which exhibit molecular weights of 69–70 kDa, or again due to a ∼50 kDa host cell protein co-purified due to formation of a disulfide bond with the Fab.

SEC-HPLC was utilized to further evaluate the monomer homogeneity and potential oligomerization of the Fab variants as illustrated in Figure 3, supplementing the SDS-PAGE findings. A predominant monomer peak was consistently observed at approximately 2.7 min across all variants, indicating the primary presence of monomeric Fab. An additional peak was detected around 2.2–2.3 min for all variants but not wild type. The height of this peak increased progressively along the truncation series from very small (<0.2 mAU above baseline) for 228TGA to slightly larger (0.5 mAU above baseline) for 223TGA, but then increased notably to 1.2 mAU above baseline for 222TGA and then 8 mAU above baseline for the 221TGA variant. While the presence of larger oligomers was expected to be excluded by the guard column, this earlier eluting peak suggests the presence of small, soluble oligomers, likely resulting from mis-paired cysteines. The molecular composition of these oligomers may include Fab trimers/tetramers, as previously indicated in the SDS-PAGE analysis, possibly in conjunction with Fab dimers and mixed Fab-LC/HC species.

**Figure 3:**
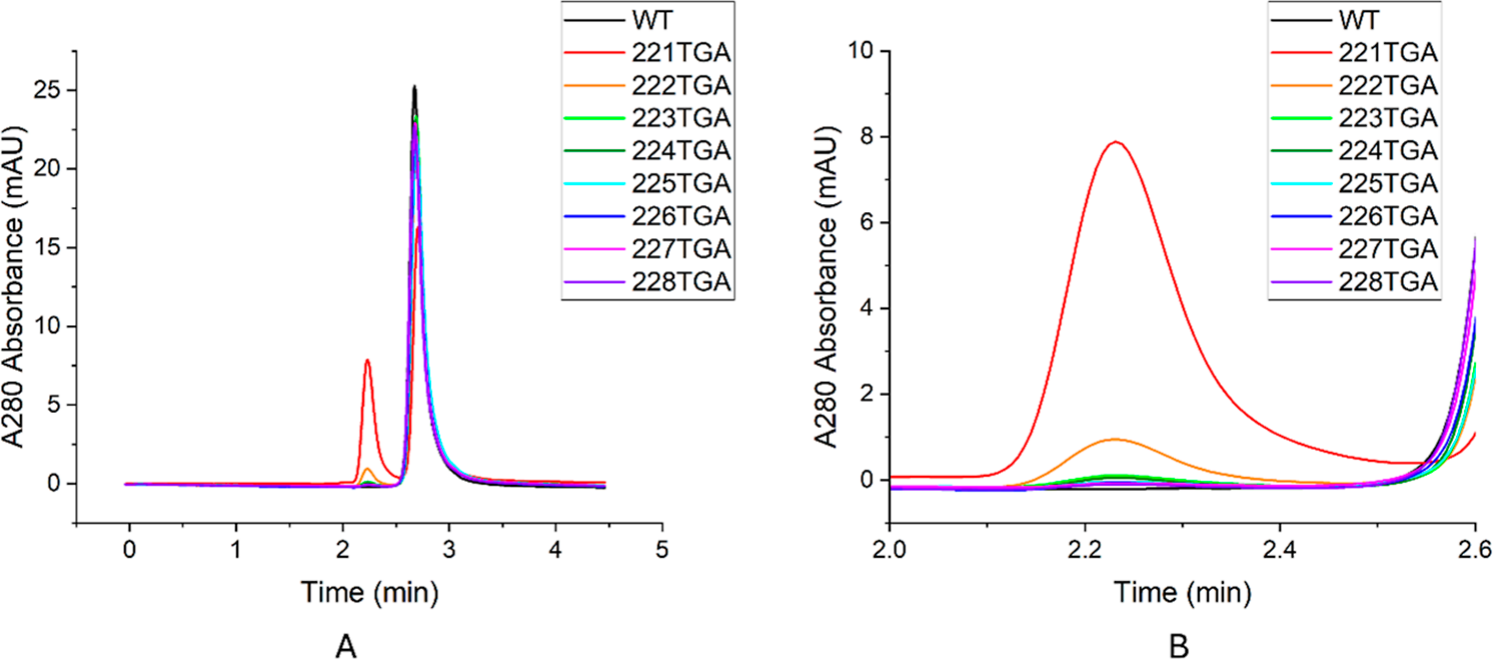
HPLC size exclusion chromatograms of the wild type (WT) and eight hinge-truncated variants from calibration. Samples were prepared at a concentration of 2 mg/mL, with injection volumes of 2 μL. Each variant is distinguished by a different color. The dimer species predominantly elutes at approximately 2.2–2.3 min, whereas the monomer elutes at around 2.7 min. Panel (B) provides a zoomed-in view of Panel (A) focusing on the elution time between 2.0 and 2.6 min.

Liquid chromatography–mass spectrometry (LC–MS) analysis was used to further elucidate the oligomerization states of the eight protein variants alongside the wild type, as shown in Figure 4. Consistently, a principal peak at approximately 46–47 kDa was observed for the wild type and all variants, confirming the predominance of the monomeric forms and indicating their good homogeneity. No other significant peaks were observed for wild type, or for the variants 222TGA to 228TGA. However, the variant 221TGA exhibited an additional peak at approximately 146 kDa, a mass coinciding nearly with the expected 140 kDa of its trimeric form and distinctly below the tetrameric form (186 kDa). The same peak was only very weakly observable above the noise for 222TGA, but not for the other variants. This suggests that the larger molecular species observed in the SDS-PAGE and SEC-HPLC analyses likely corresponded also predominantly to Fab trimers, with potential contributions from other heterogeneously sized oligomers such as dimers or antibody light chain-heavy chain (Fab-LC/HC) complexes. A comprehensive spectroscopic characterization of these findings is presented in the Supporting Information.

**Figure 4:**
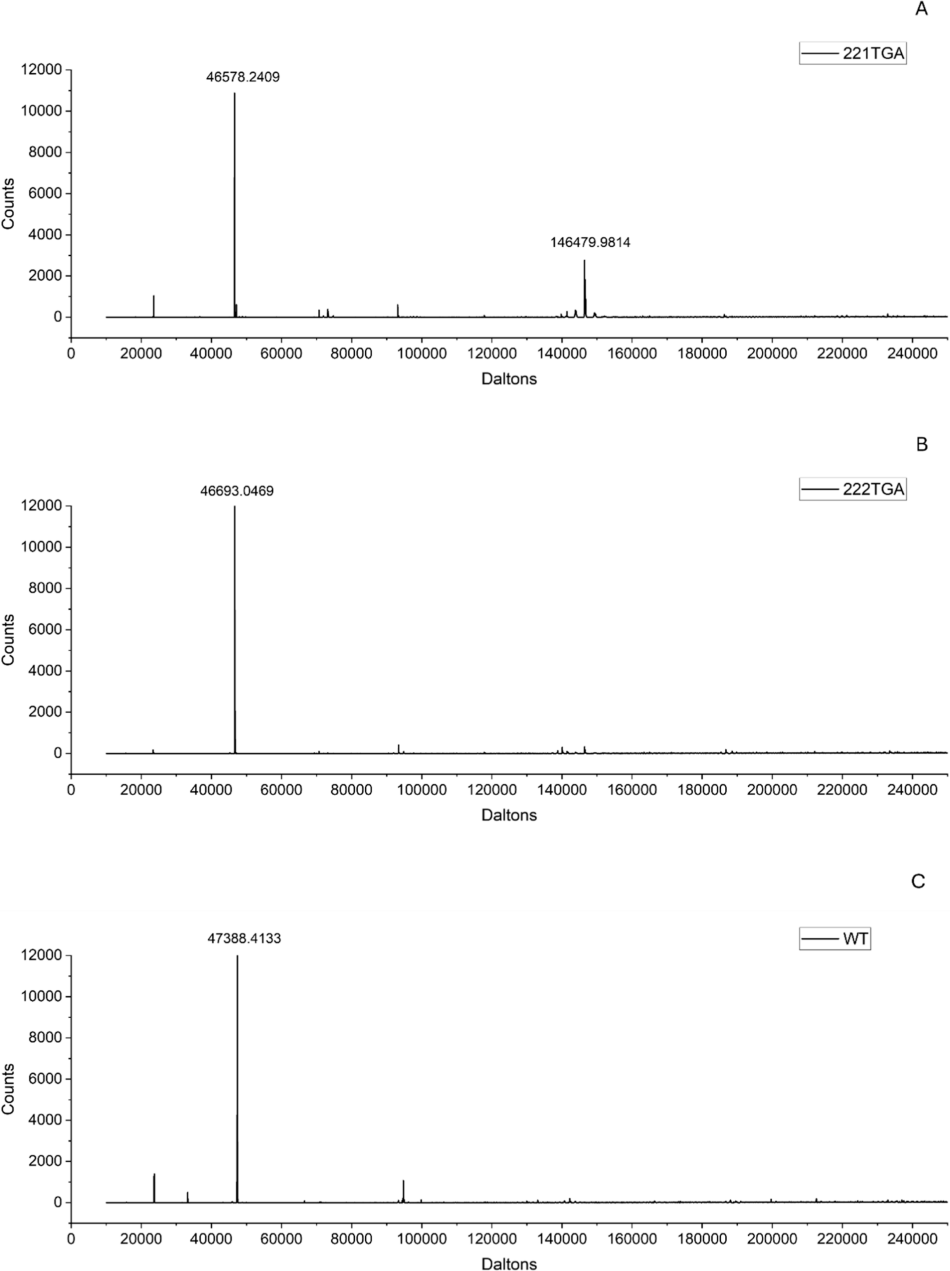
Mass spectroscopy analysis of the variants 221TGA (A), 222TGA (B) and wild type (C). Background signals were subtracted prior to deconvolution of the data. The complete data set for all the variants can be found in the Supporting Information.

### Thermal Stability Analysis

The thermal stability of the Fab variants was assessed from changes in the barycentric mean (BCM) of intrinsic fluorescence spectra as the temperature was increased. The Fab molecules were formulated in 20 mM sodium citrate, pH 4, 155 mM NaCl (200 mM total ionic strength) to ensure consistency with previous aggregation studies of the same Fab.^23^ For the majority of the samples, thermal transitions were adequately described by van’t Hoff two-state models (eq 1). However, for the variants 221TGA and 222TGA, a more complex three-state model was necessary (eq 2), presumably resulting from denaturation of the oligomeric states identified by the SDS-PAGE, SEC and LC–MS analyses above.

Specifically, the BCM data for 221TGA showed two distinct melting temperatures (*T*_m1_ and *T*_m2_) which were accurately modeled (residual sum of squares = 0.40) by the three-state model in one of the triplicate measurements (Figure 5C), suggesting a well-fitted model, but overfitted the weaker second transition in the other two replicas (Figure 5A,B). For 222TGA, the three-state model overfitted for replica 1, fitted poorly for replica 2 (residual sum of squares = 7.6), but fitted well for replica 3 (residual sum of squares = 1.0) (Figure 5D–F). By contrast, the two-state model gave a single *T*_m_ value centered on the first transition, though with an uncertain upper baseline fit. For both 221TGA and 222TGA, we retained the data from both fitting methods for comparison to other variants as their fitting statistics (residual sum of squares = 0.7–2.5) were still improved compared to the three-state fits. Detailed BCM spectral data and associated fitting statistics for all Fab variant replicas are provided in the Supporting Information.

**Figure 5:**
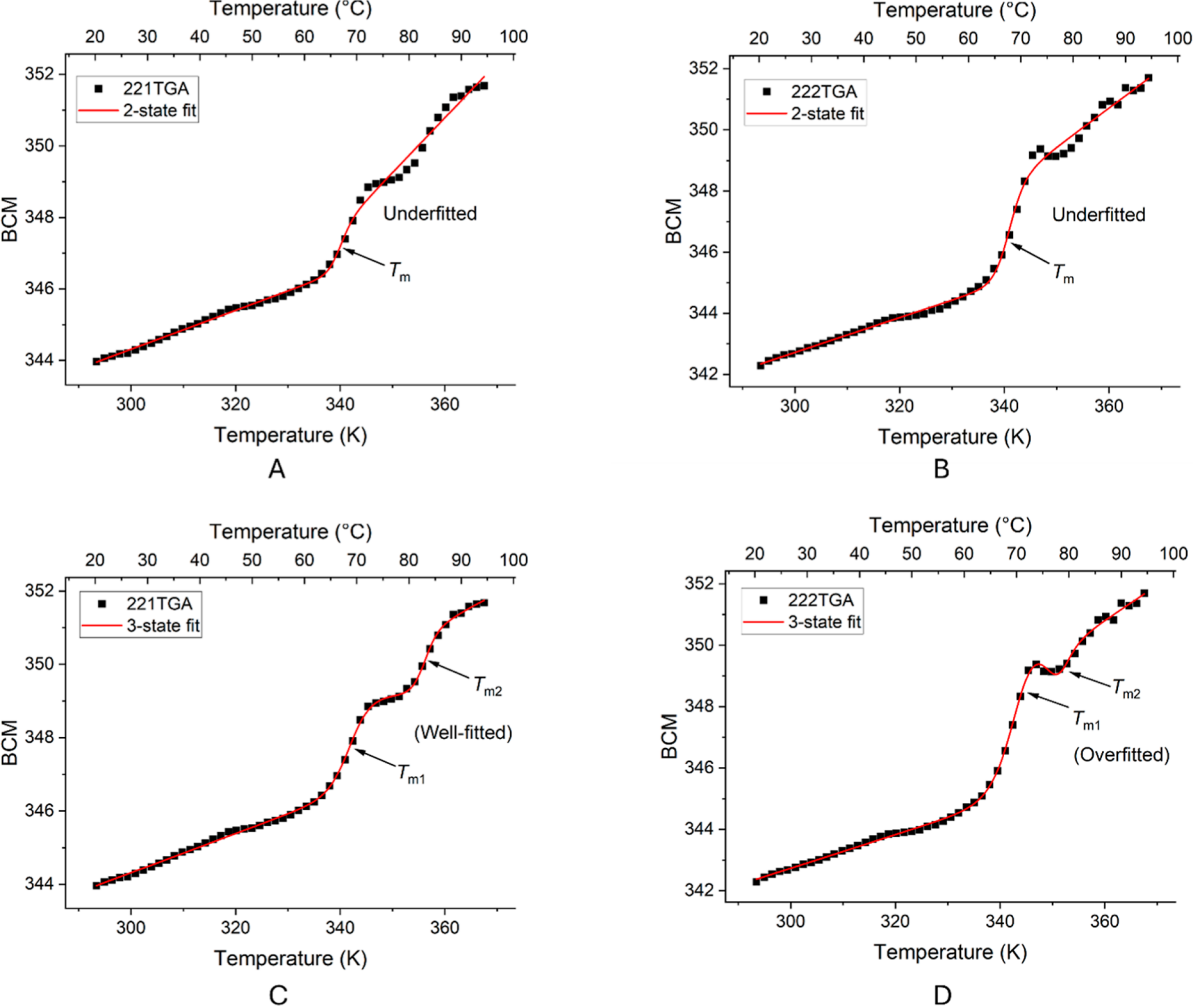
Barycentric mean (BCM) of intrinsic fluorescence spectra across temperature gradients for variants 221TGA (A&C) and 222TGA (B&D), fitted to van’t Hoff thermal transitions. The black squares represent actual measurements while the red lines indicate model fittings using two-state (top, A,B) and three-state (bottom, C,D) equations. The transition temperatures are marked with arrows. Comments on the fitting quality are included to assess the adequacy of the model fit for each scenario.

Figure 6 presents the melting temperatures (*T*_m_) and corresponding van’t Hoff entropy changes (Δ*S*_vh_), calculated using the two-state model for all variants, in addition to the three-state model for only 221TGA and 222TGA. The *T*_m_ values for most variants closely approximated that of the wild type, ranging from 69.0 to 70.5 °C. Notably, variants 221TGA and 222TGA exhibited slightly lower *T*_m_ values for the two-state model, between 66.8 and 67.6 °C, likely as a result of part of the signal change resolving out to give a separate second transition at high temperature. The *T*_m_ values from the three-state model for these particular variants were 68.1 and 82.8 °C for 221TGA, and 72.3 and 77.3 °C for 222TGA.

**Figure 6:**
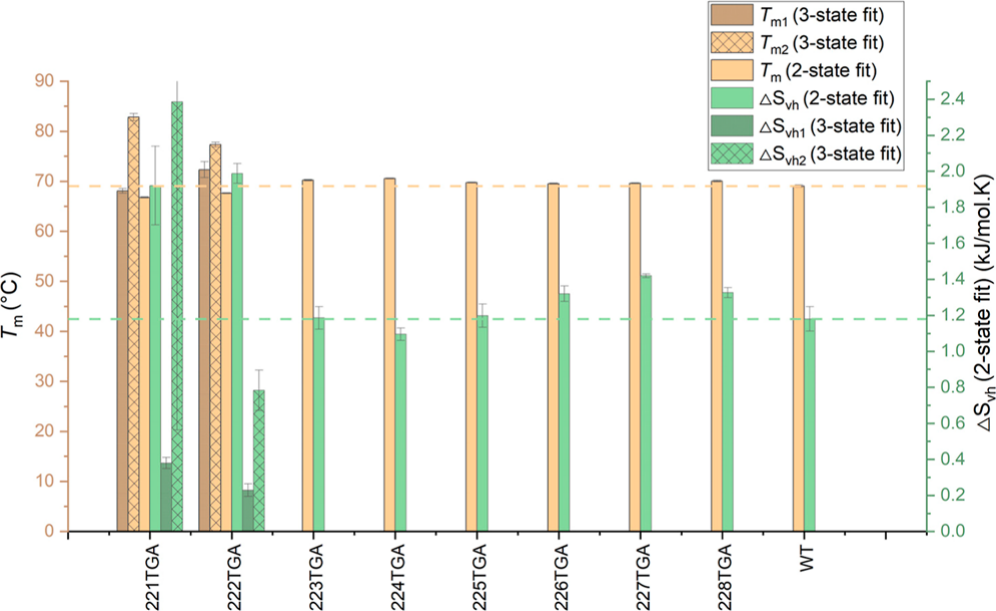
Melting temperature (*T*_m_, orange bars) and van’t Hoff entropy change (Δ*S*_vh_, green bars) for the Fab wild type (WT) and its variants labeled as 221TGA to 228TGA, fitted with a two-state thermal transition equation. For the 221TGA and 222TGA variants, values from a three-state fit are included. Dashed reference lines indicate the baseline values for WT, facilitating direct comparison of each variant’s stability relative to the WT.

Δ*S*_vh_ values can be used to monitor conformational flexibility, with elevated values suggesting a decrease in conformational flexibility within the native state, potentially leading to diminished aggregation tendencies by reducing the population of near-native, aggregation-prone conformers. Significant variations in Δ*S*_vh_ were observed among the variants. Notably, the variants 226TGA, 227TGA, and 228TGA exhibited increases of 12–20% compared to wild type. Variants 221TGA and 222TGA showed much larger (63–68%) increases in Δ*S*_vh_ compared to wild type based on two-state fitting. This reflects an apparently decreased conformational flexibility although this is also affected by the emergence of a second transition. The three-state fitting led to much lower values when comparing only their first transitions, but it is not really possible to compare these directly to the simpler two-state transitions observed for the wild type and other variants.

Static light scattering (SLS) measurements were conducted to rapidly assess the aggregation of various Fab variants. The SLS profiles at both 266 and 473 nm show that variants 221TGA and 222TGA experienced a pronounced increase in scattering intensity at lower temperatures compared to other variants (Figure 7A,B), indicating potential instability associated with the exposure of the LC214-HC220 disulfide bond. Conversely, variants 225TGA through 228TGA exhibited relatively higher temperatures prior to increases in SLS-266 nm signals, suggesting more favorable aggregation kinetics. Variants 223TGA and 224TGA, however, showed slightly earlier increases in their scattering intensity (Figure 7A).

**Figure 7:**
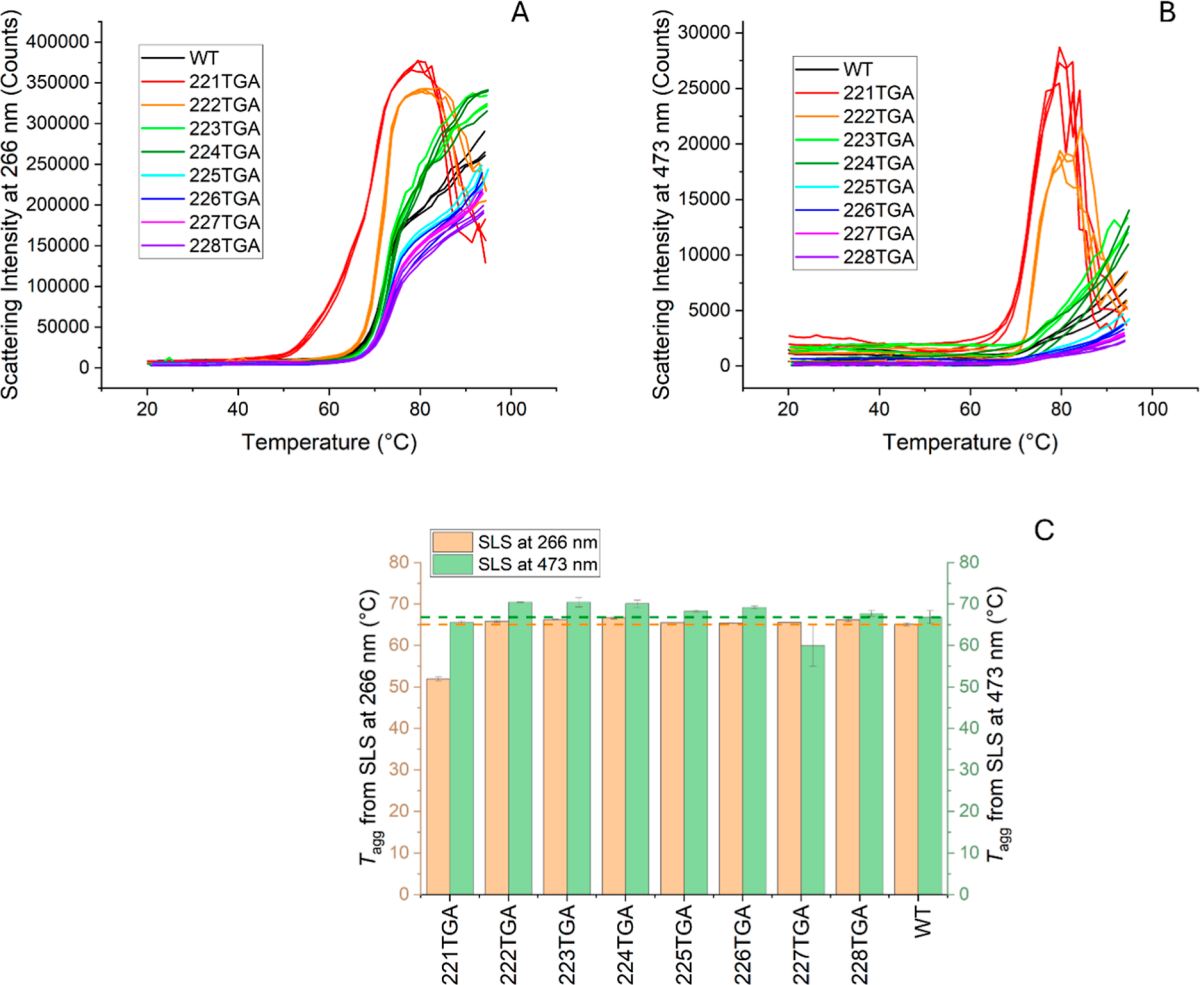
Static light scattering analysis of Fab variants. Panels (A) and (B) display the static light scattering (SLS) intensity profiles for the Fab wild type (WT) and its variants at 266 and 473 nm wavelengths, respectively. Each variant is represented by a distinct color with three repeat measurements in the same color. The corresponding aggregation onset temperatures (*T*_agg_) are summarized in panel (C), indicating *T*_agg_ values at 266 nm (orange bars) and 473 nm (green bars), with standard error of the mean (SEM) represented by error bars for each data point. Dashed reference lines indicate the baseline values for WT, facilitating direct comparison of each variant’s stability relative to the WT.

The SLS data at 473 nm indicated that the 221TGA and 222TGA variants began to form larger particles at lower temperatures, with a marked increase in scattering intensity after 70 °C. In contrast, the remaining variants initiated particle formation at slightly higher temperatures of 80 °C, and demonstrated lower scattering intensity. The differentiation among the variants based on scattering intensity at 473 nm was minimal, likely due to the reduced sensitivity of this wavelength to smaller aggregate forms (Figure 7B). Moreover, the *T*_agg_ values derived from the Uncle software (Figure 7C) did not always reflect the stability ranking observable directly from the SLS spectra profiles. These values primarily capture the point at which scattering intensity increased, which was relatively similar across the variants other than for 221TGA.

### Aggregation Kinetics of Hinge-Truncated Variants of Fab Analyzed by SEC-HPLC

The aggregation kinetics of WT Fab and the hinge-truncated variants were quantitatively analyzed by monomer retention using SEC-HPLC over time as shown in Figure 8A. Measurements for triplicate samples are shown alongside their curves fitted independently to eq 3. The aggregation kinetic constants for these variants and WT are shown in Figure 8B. Table 1 summarizes the kinetic parameters and thermal stability data.

**Figure 8:**
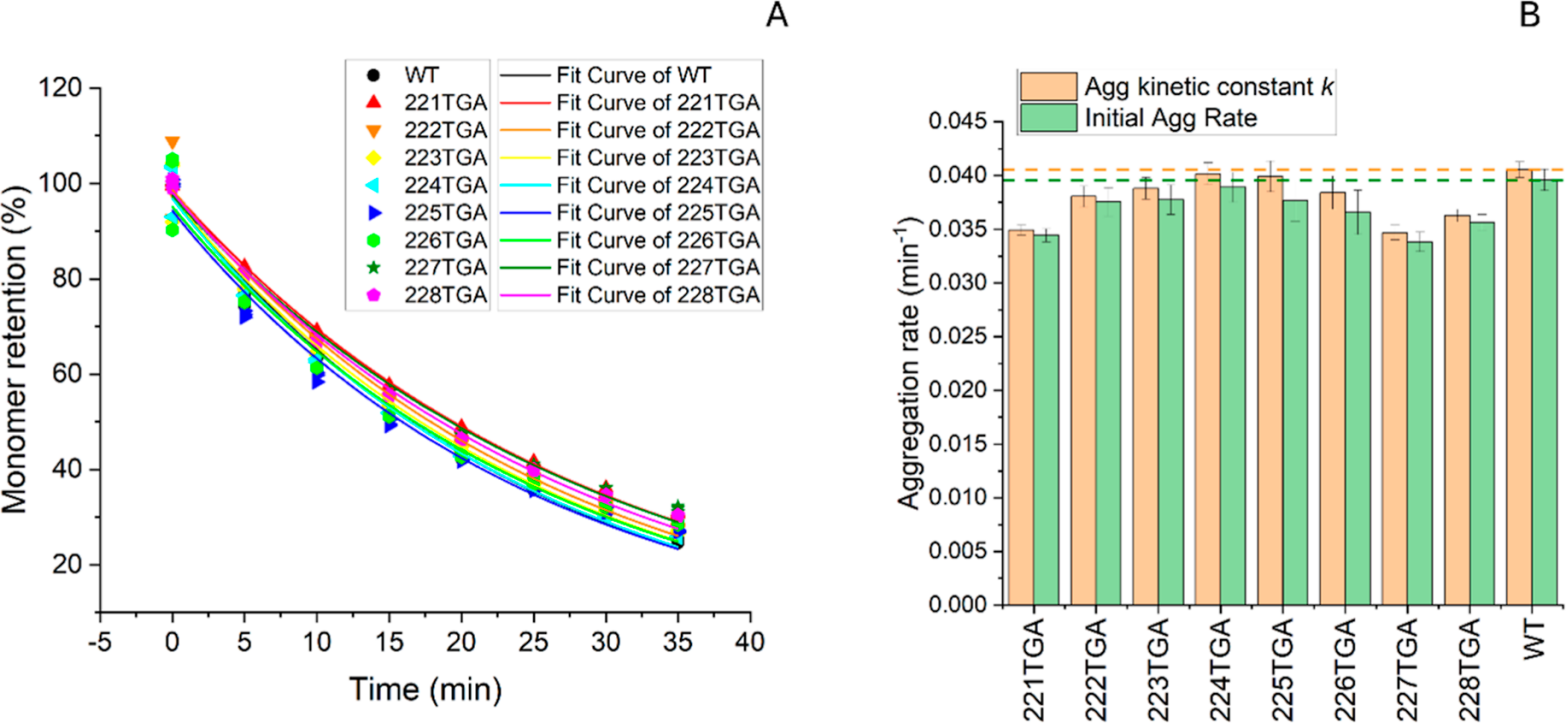
Aggregation kinetics as measured by SEC-HPLC. Panel (A) the monomer retention over time for the wild type (WT) and eight hinge-truncated variants. All three replicas are shown for each variant at each time-point. For each variant, a single monomer retention curve is fitted to the combined replica data (24 data points) using eq 3 to derive the kinetic constant, *k*, modeled reaction coefficient, *A*, and associated standard fitting errors. Unique colors and symbols represent each variant. Panel (B) aggregation rate constant *k* and initial aggregation rate derived from eq 3 and *A* × *k* respectively. Reference lines for WT are included for comparison. Error bars represent standard errors based on parameter fitting errors.

**Table 1:** Data Summary of Thermal Stability and Aggregation Kinetics for the Fab Wild Type and Variants^a^

	*T*_m_ (°C)		Δ*H*_vh_ (kJ/mol)		Δ*S*_vh_ (kJ/mol K)		aggregation rate constant		modelled coefficient *A*	initial aggregation rate	
variant ID	Ave	SEM	Ave	SEM	Ave	SEM	*k* (min^–1^)	SE		rate (min^–1^)	relative to WT
221TGA	66.8	0.13	653	74	1.9	0.22	0.0349	0.0005	0.986	0.0344	–13.0%
222TGA	67.6	0.11	677	18	2.0	0.05	0.038	0.001	0.987	0.0376	–5.2%
223TGA	70.2	0.15	407	22	1.2	0.06	0.039	0.001	0.973	0.0378	–4.6%
224TGA	70.5	0.09	376	12	1.1	0.03	0.040	0.001	0.969	0.0389	–1.7%
225TGA	69.7	0.09	411	22	1.2	0.06	0.0399	0.0014	0.943	0.0377	–4.9%
226TGA	69.5	0.1	452	15	1.3	0.04	0.0384	0.0016	0.952	0.0366	–7.6%
227TGA	69.6	0.08	486	4	1.4	0.01	0.0347	0.0007	0.975	0.0338	–14.5%
228TGA	70.0	0.16	455	9	1.3	0.03	0.0363	0.0006	0.981	0.0356	–10.0%
WT	69.0	0.24	404	24	1.2	0.07	0.0406	0.0007	0.976	0.0396	0.0%

a The melting temperature (*T*_m_), enthalpy change of unfolding (Δ*H*_vh_), and entropy change of unfolding (Δ*S*_vh_) for individual repeats were determined by fitting barycentric mean (BCM) intrinsic fluorescence spectra to a two-state transition model. Averaged (Ave) and standard errors of the mean (SEM) were from triplicate measurements. Aggregation kinetics were analysed by fitting the combined monomer retention data for triplicates to eq 3 to determine the rate constant *k* and modelled coefficient A for each variant, and associated standard error (SE) from fitting.

Generally, all variants followed first-order kinetics, exhibiting lower aggregation rates than WT. Notably, the shortest hinge variant, 221TGA, showed a 13.0% reduction in aggregation rate. As the hinge length increased stepwise for the next four residues, the aggregation rate approached that of WT, with 224TGA showing a minimal reduction of 1.7%. Further extension of the hinge length resulted in a continued decrease in aggregation rate, with 227TGA and 228TGA displaying reductions of 14.5% and 10.0%, respectively.

The overall kinetics of aggregation were maximally decreased by 14.5%, highlighting the effectiveness of optimizing hinge length as a strategy to mitigate aggregation. A correlation between aggregation rate and the van’t Hoff entropy change (Δ*S*_vh_), in Figure 9, demonstrates a strong linear relationship (*R* ^2^ = 0.936) when excluding the outlier variants 221TGA and 222TGA that had two thermal melting transitions instead of one. Thus, reduction in the overall native protein flexibility led to slower aggregation. This result, in which aggregation kinetics correlated with Δ*S*_vh_ while the *T*_m_ values remained unchanged was also observed in our previous work in which stabilizing mutations were targeted to regions with the most flexible structure in the C_H_ and C_L_ domains.^26,27^ Thus, as proposed in that work, it is likely that flexibility in the hinge region and surrounding structure plays a role in promoting aggregation of A33Fab, potentially through partial unfolding to increase solvent exposure of an aggregation prone region. Stabilizing mutations, or the hinge truncations of the present study, can be used to minimize flexibility and partial unfolding of local regions that therefore minimize aggregation.

**Figure 9:**
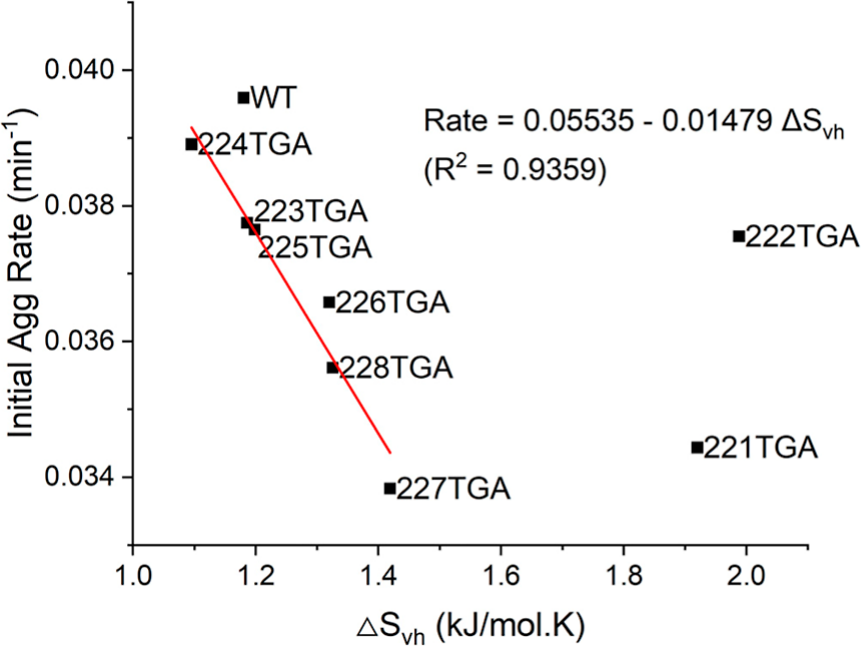
Linear regression analysis of the initial aggregation rate versus van’t Hoff entropy change (Δ*S*_vh_) for various Fab variants. The red line represents the linear fit, excluding outliers 221TGA and 222TGA.

The increased Δ*S*_vh_ in the outliers 221TGA and 222TGA was likely to have been linked to the observed increase in oligomerization within the starting material, resulting from disulfide bond formation between the exposed cysteine residues. This would complicate their aggregation behavior by involving both covalent oligomer formation and noncovalent monomer aggregation, which skews their data from the linear trend. The impact of aggregates on unfolding cooperativity, measured as Δ*S*_vh_ (or Δ*H*_vh_), is well-known.^28^

Despite the increased presence of oligomers in the 221TGA and 222TGA starting materials, these variants showed significant reductions in aggregation rate. This indicates that the subsequent aggregation of Fab monomers under the forced degradation conditions was independent of the initial oligomer formation. Regardless, the instability of exposed cysteine residues, particularly in 221TGA, could lead to posttranslational modifications, complicating downstream purification processes and posing regulatory challenges. Conversely, variants 227TGA and 228TGA demonstrated optimal characteristics for reducing aggregation with no initial oligomer formation. Notably, the 14.5% reduction in aggregation observed with 227TGA represents an enhancement over the previous mutational optimization, which achieved improvements in the range of 5–11%.^26^

Overall, the most extensive truncation of the A33 Fab hinge in 221TGA, and less so for 222TGA, led to more oligomers in the initially purified protein as detected by SDS-PAGE, SEC and LC–MS, and also indirectly through SLS. Oxidising versus reducing SDS-PAGE indicated that these were formed through intermolecular disulfide bonds, most likely as a result of greater solvent exposure of the cysteines at residues LC-214 and HC-220. The initial presence of these oligomers did not appear to directly influence subsequent aggregation kinetics in the forced degradation conditions, but they did alter the unfolding cooperativity of the 221TGA and 222TGA variants as evidenced by their increased Δ*S*_vh_ for unfolding and the emergence of a second thermal denaturation transition.

The other variants did not contain significant oligomers in the starting material, and the progressive truncation from 228TGA to 223TGA led to changes in aggregation kinetics under forced degrdation conditions that were nonlinear with hinge length, but that did correlate very well with their Δ*S*_vh_ for unfolding, while their *T*_m_ values remained essentially unchanged. This behavior was observed previously with point mutations within the hinge region, and highlights that increased hinge region flexibility leads to faster aggregation kinetics.

Variants 228TGA and 227TGA were the most stable kinetically under forced degradation conditions, and also contained minimal oligomer content in the starting material. This indicates that the hinge was still long enough to protect the nearby cysteine residues from intermolecular disulfide formation, and also that the removal of the two terminal alanine residues was important for suppressing aggregation kinetics. It is possible that these two residues were themselves involved in enhancing protein–protein interactions that promote aggregation, or that their increased structural flexibility induced further structural dynamics in the local region that could promote aggregation.

## Conclusion

In this study, we explored the effects of hinge truncation on the thermal stability and aggregation propensity of an antibody Fab fragment. By engineering eight Fab variants with progressively shortened hinge regions, we demonstrated that truncating the hinge region can significantly enhance the thermal stability and reduce aggregation. Notably, the 227TGA variant showed the most promising results, with a 14.5% reduction in aggregation rate compared to the wild type, while maintaining comparable thermal stability. These findings suggest that hinge truncation is a viable strategy for improving the developability of therapeutic antibody fragments, particularly those prone to stability issues.

Future research should aim to evaluate the impact of hinge truncations on other measures of stability (agitation, light stress, packaging interactions), alongside in vivo efficacy and pharmacokinetics, would also provide valuable insights in future. It would also be useful in future to demonstrate the generalizability of hinge truncation to maximize stability and minimize aggregation across a broader range of antibody fragments. Overall, hinge truncation represents a promising avenue to explore for other antibody fragments under development as therapeutics or diagnostics.

## Supplementary Material



## Notes

Fab amino acid sequence; Mass spectra of all Fab variants; Table of mass spectrometry data for all variants; Table to compare theoretical and experimental massess of variants; Two-state and three-state fits for differential scanning fluorescence thermograms; SEC-HPLC retention times for all Fab variants (PDF)
