# Supplementary material for: Hinge Truncation to Improve Aggregation Kinetics and Thermal Stability of an Antibody Fab Fragment
Source: Mol Pharm. 2025 Aug 7;22(9):5389–99. doi: 10.1021/acs.molpharmaceut.5c00358 (PMC12406249; doi:10.1021/acs.molpharmaceut.5c00358)
Supplement: Supplementary file 1 [file mp5c00358_si_001.pdf]

Supplementary Information (SI) for

# Hinge truncation to improve aggregation kinetics and thermal stability of an antibody Fab fragment

*Cheng Zhang[1], Kersti Karu[2], Paul Dalby[1*]*

[1]Department of Biochemical Engineering, University College London, Gower Street, London, WC1E 6BT

[2]Department of Chemistry, University College London, 20 Gordon Street, London, WC1H 0AJ

*Correspondence email: p.dalby@ucl.ac.uk

# 1 Fab amino acid sequence

SI Table 1. The amino acid and DNA sequences for the Fab light and heavy chain. To truncate the 1-8 residues at the end of hinge region, "TGA" stop codon was introduced to replace the corresponding ones (highlighted in red and underline), resulting variant 228TGA, 227TGA, 226TGA, 225TGA, 224TGA, 223TGA, 222TGA, 221TGA, respectively.

| | | |
|---|---|---|
| Light chain (LC) | Amino acid | DIQMTQSPSSLSASVGDRVTITCKASQNVRTVVAWYQQKPGKAPKTLIYLASNRHTG<br>VPSRFSGSGSGTDFTLTISSLQPEDFATYFCLQHWSYPLTFGQGTKVEIKRTVAAPS<br>VFIFPPSDEQLKSGTASVVCLLNNFYPREAKVQWKVDNALQSGNSQESVTEQDSKD<br>STYSLSSTLTLSKADYEKHKVYACEVTHQGLSSPVTKSFNRGEC |
| | DNA | GATATCCAGATGACTCAGAGTCCAAGTAGTCTCAGTGCTAGTGTAGGTGATAGG<br>GTAACTATCACTTGTAAGGCCAGTCAGAATGTTCGTACTGTTGTAGCCTGGTATC<br>AGCAGAAACCAGGTAAAGCCCCAAAAACTCTCATCTATTTGGCCTCCAACCGGCA<br>CACTGGAGTACCATCTAGATTCAGTGGTAGCGGTAGTGGTACTGATTTCACTCTG<br>ACTATCAGTAGTCTCCAGCCAGAAGATTTCGCCACTTACTTCTGCCTACAACATT<br>GGAGTTATCCTCTCACGTTCGGTCAGGGTACTAAAGTAGAAATCAAACGTACGGT<br>AGCGGCCCCATCTGTCTTCATCTTCCCGCCATCTGATGAGCAGTTGAAATCTGGA<br>ACTGCCTCTGTTGTGTGCCTGCTGAATAACTTCTATCCCAGAGAGGCCAAAGTAC<br>AGTGGAAGGTGGATAACGCCCTCCAATCGGGTAACTCCCAGGAGAGTGTCACAG<br>AGCAGGACAGCAAGGACAGCACCTACAGCCTCAGCAGCACCCTGACGCTGAGC<br>AAAGCAGACTACGAGAAACACAAAGTCTACGCCTGCGAAGTCACCCATCAGGGC<br>CTGAGCTCACCAGTAACAAAAAGTTTTAATAGAGGGGAGTGT |
| Heavy chain (HC) | Amino acid | EVQLVESGGGLVQPGGSLRLSCAASGFAFSTYDMSWVRQAPGKGLEWVATISSGG<br>SYTYYLDSVKGRFTISRDSSKNTLYLQMNSLRAEDTAVYYCAPTTVVPFAYWGQGTL<br>VTVSSASTKGPSVFPLAPSSKSTSGGTAALGCLVKDYFPEPVTVSWNSGALTSGVH<br>TFPAVLQSSGLYSLSSVVTVPSSSLGTQTYICNVNHKPSNTKVDKKVEPKSCD K T H<br>T S A A |
| | DNA | GAGGTTCAGCTGGTGGAGTCTGGAGGAGGACTGGTGCAGCCTGGAGGATCTCT<br>GAGACTGTCTTGTGCAGCATCTGGATTCGCTTTCAGTACCTATGACATGTCTTGG<br>GTGAGACAGGCACCTGGAAAAGGACTCGAGTGGGTGGCAACCATTAGTAGTGGT<br>GGTAGTTACACCTACTATTTAGACAGTGTGAAGGGAAGATTCACAATTTCCAGAG<br>ACTCTAGCAAGAATACACTGTACCTGCAGATGAACTCTCTGCGGGCAGAGGACA<br>CTGCAGTTTACTACTGTGCACCGACTACGGTAGTCCCGTTTGCTTACTGGGGACA<br>GGGAACACTGGTGACAGTGTCTTCTGCCTCAACGAAGGGCCCATCGGTCTTCCC<br>CCTGGCACCCTCCTCCAAGAGCACCTCTGGGGGCACAGCGGCCCTGGGCTGCC<br>TGGTCAAGGACTACTTCCCCGAACCGGTGACGGTGTCGTGGAACTCAGGCGCC<br>CTGACCAGCGGCGTGCACACCTTCCCGGCTGTCCTACAGTCCTCAGGACTCTAC<br>TCCCTCAGCAGCGTGGTGACCGTGCCCTCCAGCAGCTTGGGCACCCAGACCTA<br>CATCTGCAACGTGAATCACAAGCCCAGCAACACCAAGGTCGACAAGAAAGTTGA<br>GCCCAAATCTTGTGAC AAA ACT CAC ACA AGC GCC GCG |

# 2 Mass Spectroscopy

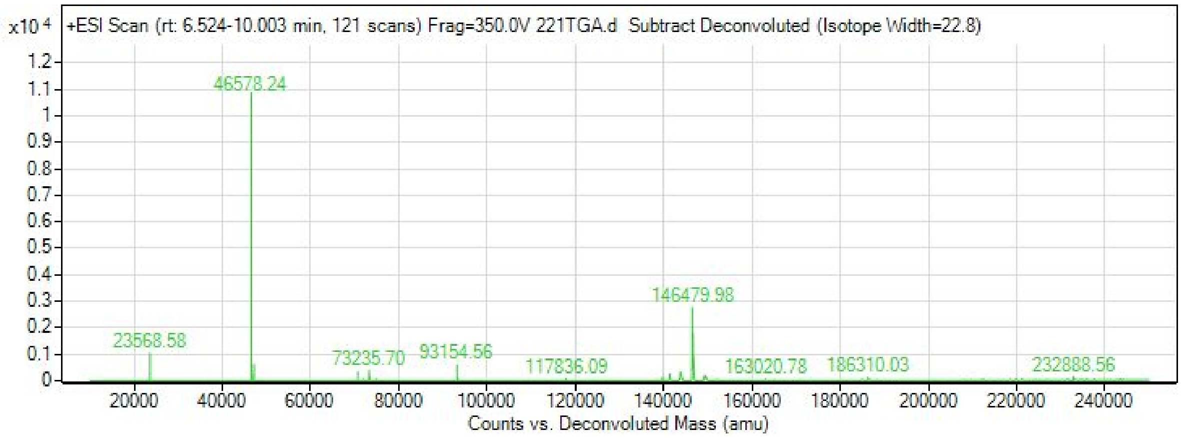


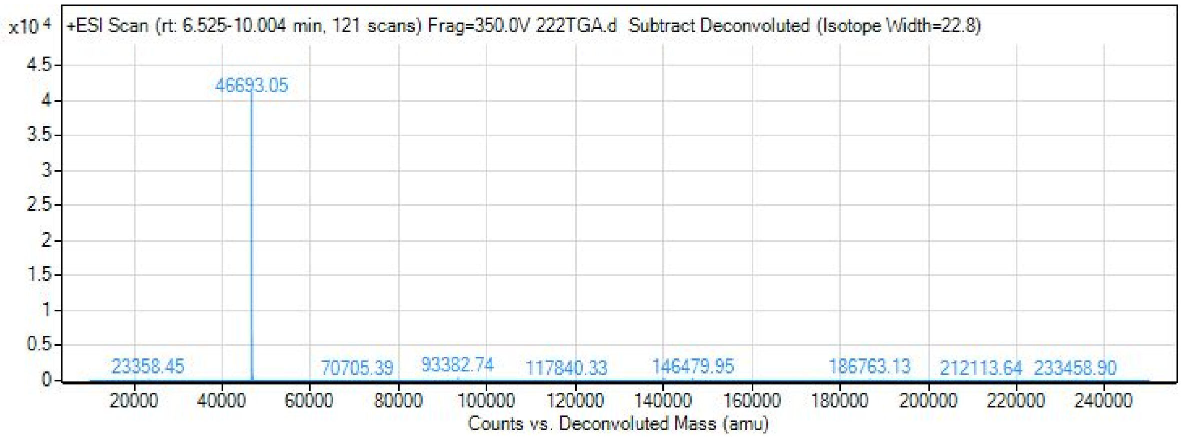


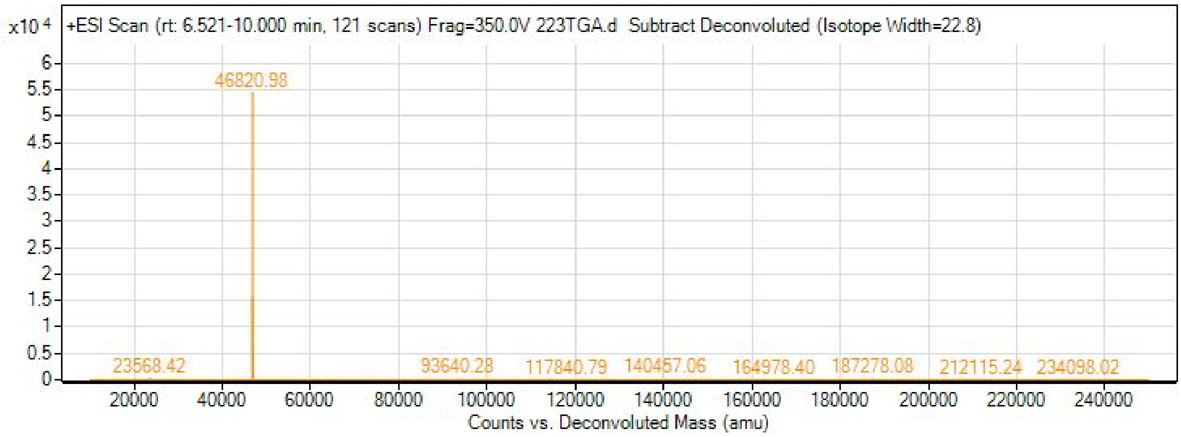


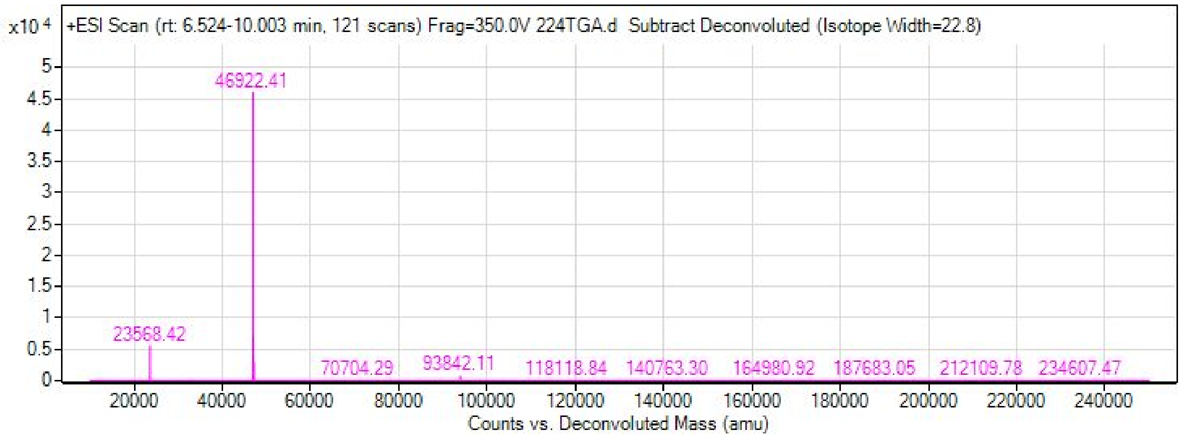

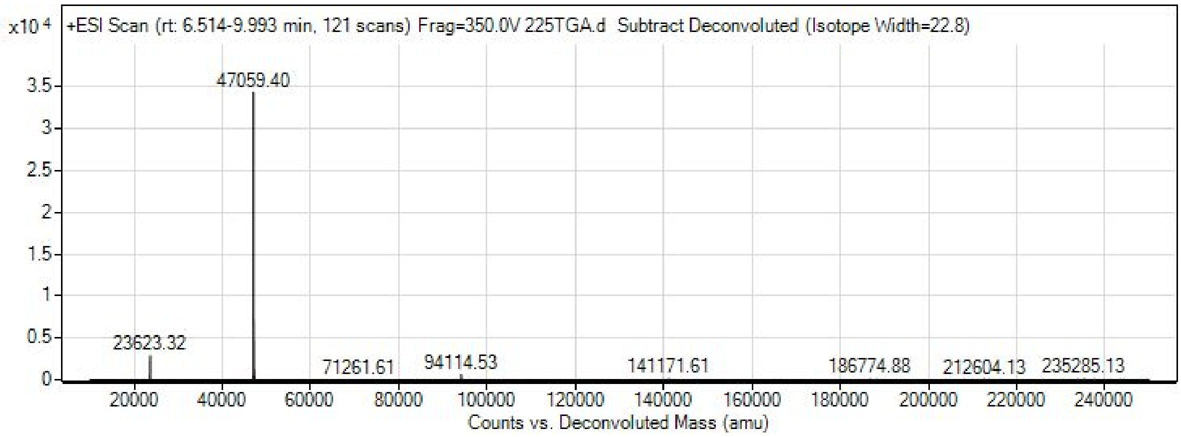
x10 4
+ESI Scan (rt: 6.514-9.993 min, 121 scans) Frag=350.0V 225TGA.d Subtract Deconvoluted (Isotope Width=22.8)
47059.40
23623.32
71261.61
94114.53
141171.61
186774.88
212604.13
235285.13
Counts vs. Deconvoluted Mass (amu)

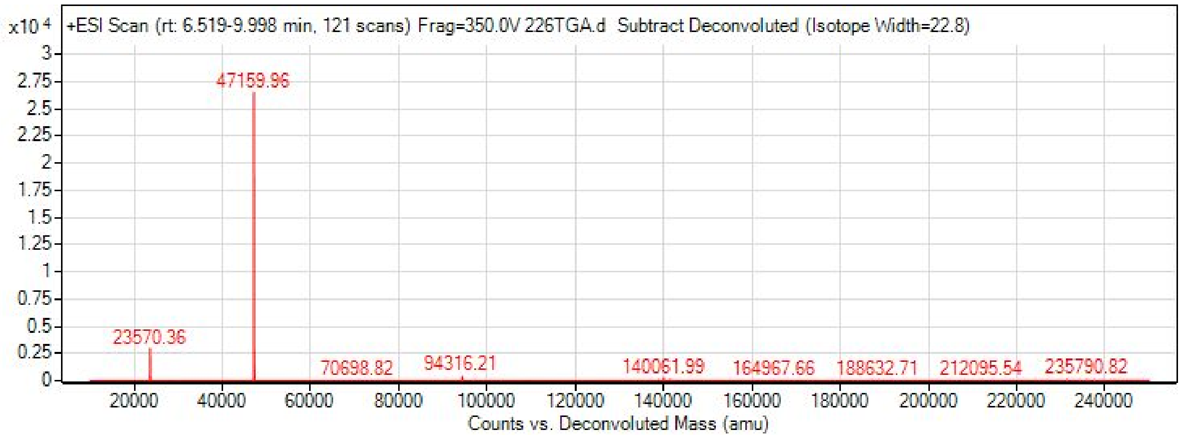
x10 4
+ESI Scan (rt: 6.519-9.998 min, 121 scans) Frag=350.0V 226TGA.d Subtract Deconvoluted (Isotope Width=22.8)
47159.96
23570.36
70698.82
94316.21
140061.99
164967.66
188632.71
212095.54
235790.82
Counts vs. Deconvoluted Mass (amu)

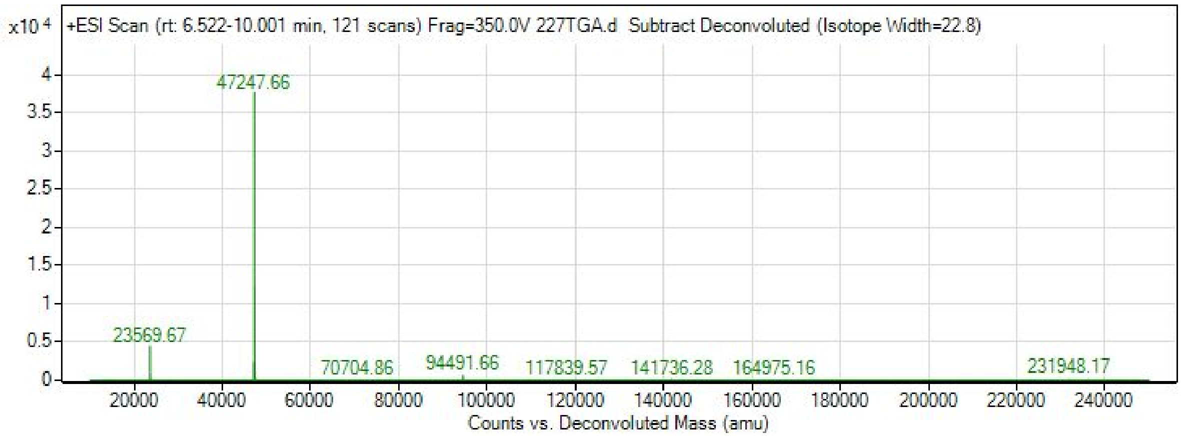
x10 4
+ESI Scan (rt: 6.522-10.001 min, 121 scans) Frag=350.0V 227TGA.d Subtract Deconvoluted (Isotope Width=22.8)
47247.66
23569.67
70704.86
94491.66
117839.57
141736.28
164975.16
231948.17
Counts vs. Deconvoluted Mass (amu)

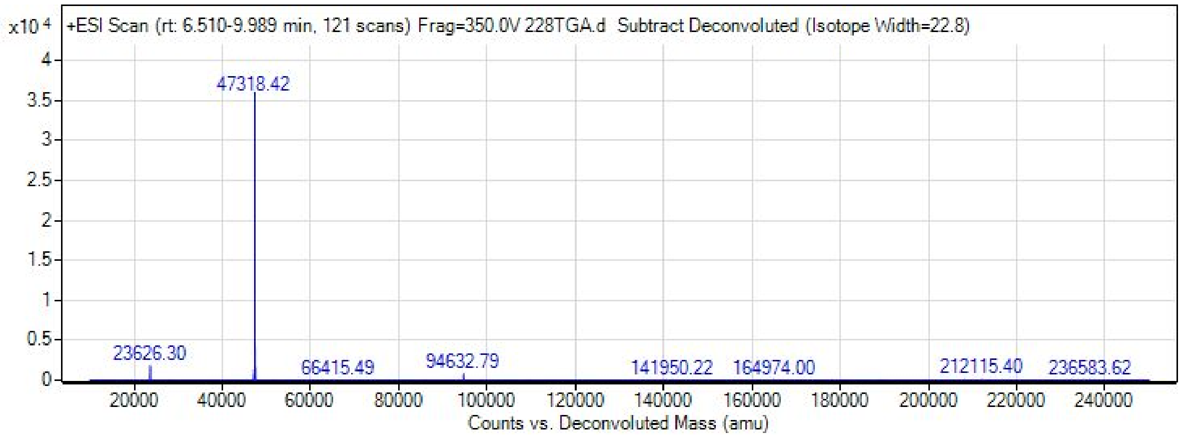
x10 4
+ESI Scan (rt: 6.510-9.989 min, 121 scans) Frag=350.0V 228TGA.d Subtract Deconvoluted (Isotope Width=22.8)
47318.42
23626.30
66415.49
94632.79
141950.22
164974.00
212115.40
236583.62
Counts vs. Deconvoluted Mass (amu)

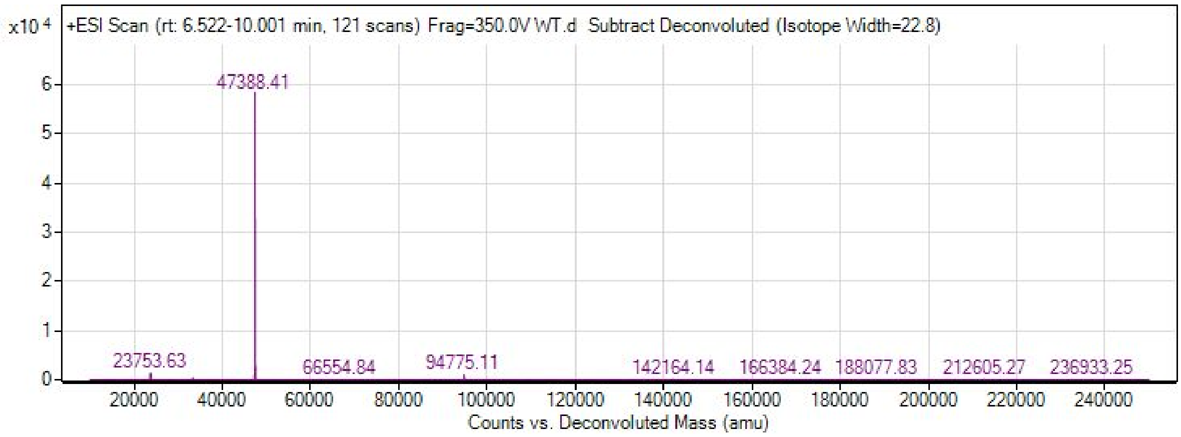


SI Figure 1. Complete mass spectroscopy analysis for all variants and the wild type (WT). Variants 221TGA through 228TGA, along with the WT, are sequentially presented from top to bottom. All spectra were processed by subtracting background signals before deconvolution.

SI Table 2. Summary of mass (Da), peak height, and compound identification for each sample as depicted in **Error! Reference source not found.**. The table presents data sequentially for variants 221TGA through 228TGA and the wild type (WT), with alternating white and grey background to distinguish between different samples.

| Sample | Mass (Da) | Height | Label |
|---|---|---|---|
| 221TGA | 46578.241 | 10758 | Compound 1 |
| 221TGA | 146479.98 | 2757 | Compound 2 |
| 221TGA | 146635.16 | 1824 | Compound 3 |
| 222TGA | 46693.047 | 40331 | Compound 1 |
| 223TGA | 46820.984 | 54591 | Compound 1 |
| 223TGA | 46951.616 | 1671 | Compound 2 |
| 224TGA | 46922.413 | 45858 | Compound 1 |
| 224TGA | 32810.646 | 183 | Compound 2 |
| 225TGA | 47059.396 | 34415 | Compound 1 |
| 226TGA | 47159.956 | 26498 | Compound 1 |
| 227TGA | 47247.655 | 37359 | Compound 1 |
| 228TGA | 47318.417 | 35652 | Compound 1 |
| WT | 47388.413 | 57890 | Compound 1 |
| WT | 33276.994 | 505 | Compound 2 |

SI Table 3. Comparison between the theoretical mass (https://web.expasy.org/compute_pi/) and measured mass (

SI Table 2) for the Fab wild type and variants.

| MW (Dalton) | 221TGA | 222TGA | 223TGA | 224TGA | 225TGA | 226TGA | 227TGA | 228TGA | WT |
|---|---|---|---|---|---|---|---|---|---|
| Theoretical Mass | 46566.25 | 46681.34 | 46809.52 | 46910.62 | 47047.76 | 47148.87 | 47235.95 | 47307.02 | 47378.10 |
| Measured Mass | 46578.241 | 46693.047 | 46820.984 | 46922.413 | 47059.396 | 47159.956 | 47247.655 | 47318.417 | 47388.413 |

# 3 Differential scanning fluorescence thermograms

## 3.1 2-state fit to changes in barycentric mean (BCM)

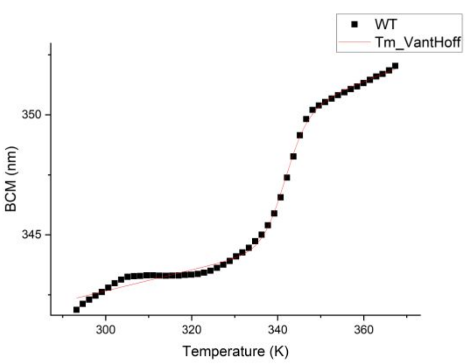


A

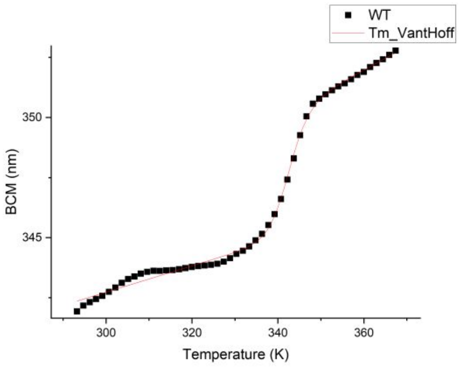


B

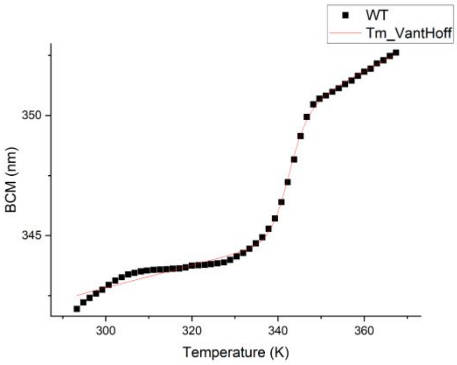


C

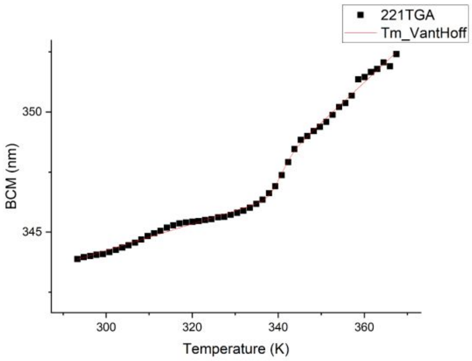


D

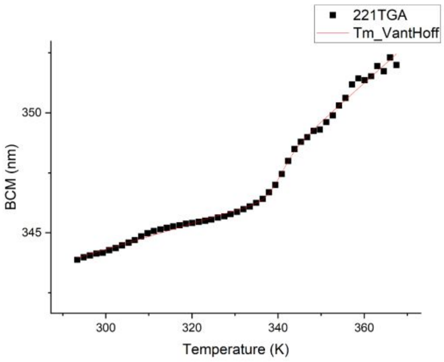


E

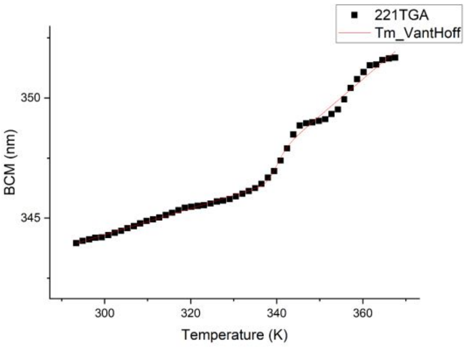


F

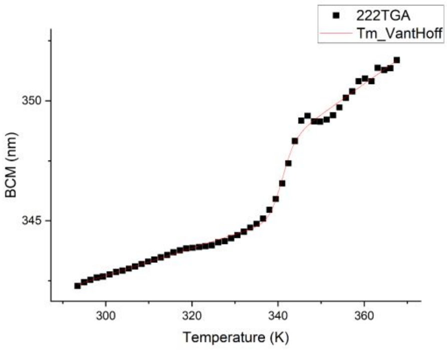


G

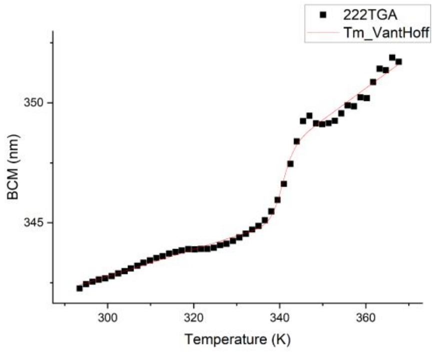


H

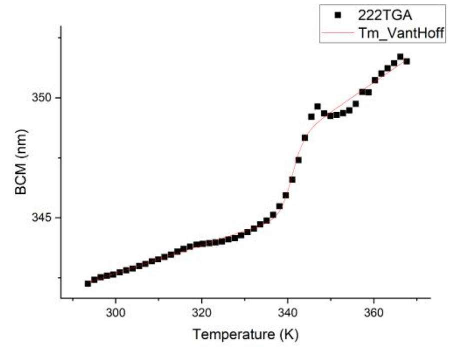
222TGA
Tm_VantHoff
BCM (nm)
Temperature (K)
345
350
300
320
340
360

I

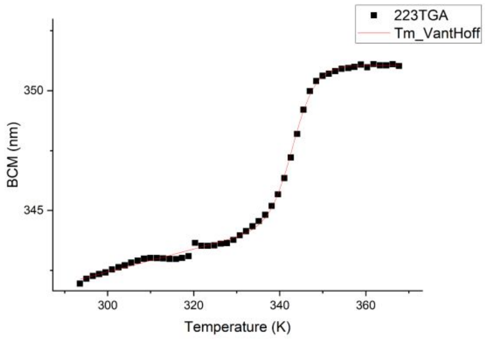
223TGA
Tm_VantHoff
BCM (nm)
Temperature (K)
345
350
300
320
340
360

J

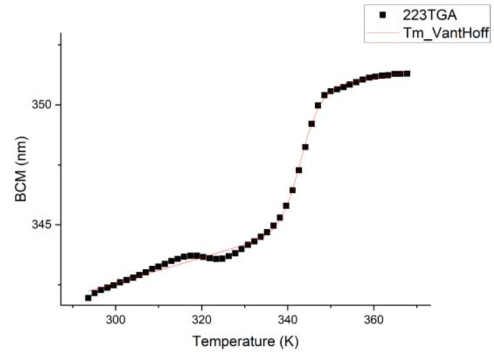
223TGA
Tm_VantHoff
BCM (nm)
Temperature (K)
345
350
300
320
340
360

K

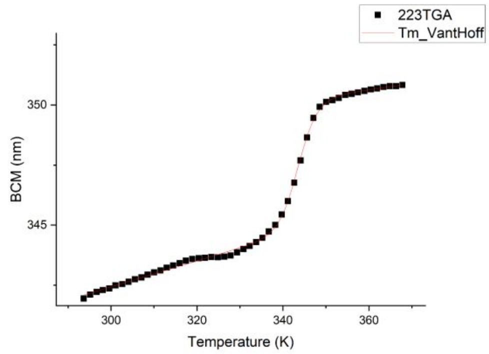
223TGA
Tm_VantHoff
BCM (nm)
Temperature (K)
345
350
300
320
340
360

L

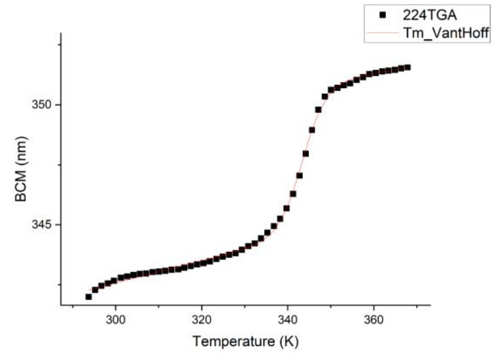
224TGA
Tm_VantHoff
BCM (nm)
Temperature (K)
345
350
300
320
340
360

M

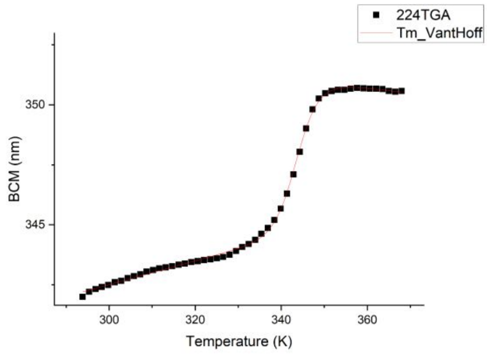
224TGA
Tm_VantHoff
BCM (nm)
Temperature (K)
345
350
300
320
340
360

N

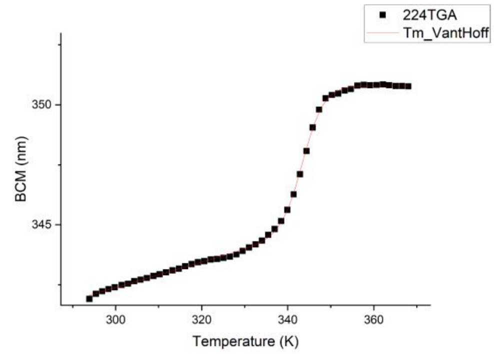
224TGA
Tm_VantHoff
BCM (nm)
Temperature (K)
345
350
300
320
340
360

O

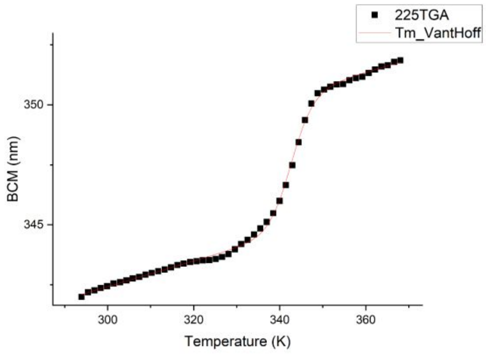
225TGA
Tm_VantHoff
BCM (nm)
Temperature (K)
345
350
300
320
340
360

P

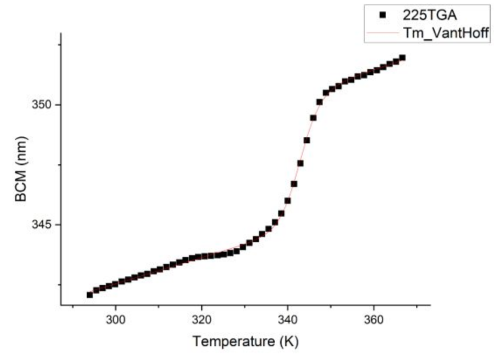
225TGA
Tm_VantHoff
BCM (nm)
Temperature (K)
345
350
300
320
340
360

Q

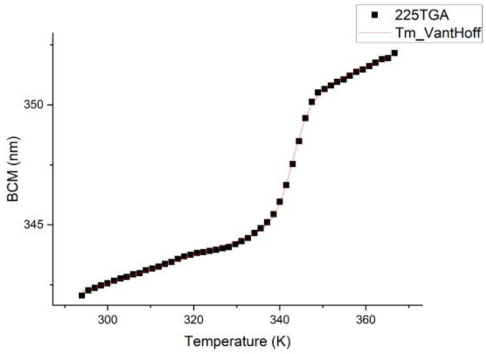
225TGA
Tm_VantHoff
BCM (nm)
Temperature (K)
345
350
300
320
340
360

R

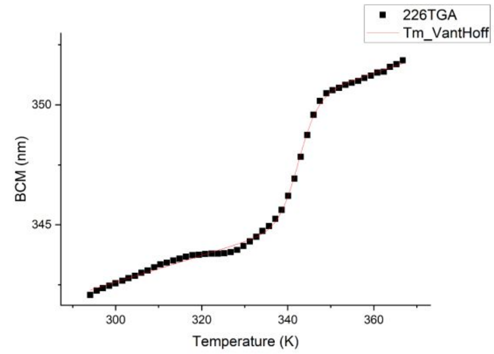
226TGA
Tm_VantHoff
BCM (nm)
350
345
300
320
340
360
Temperature (K)

**S**

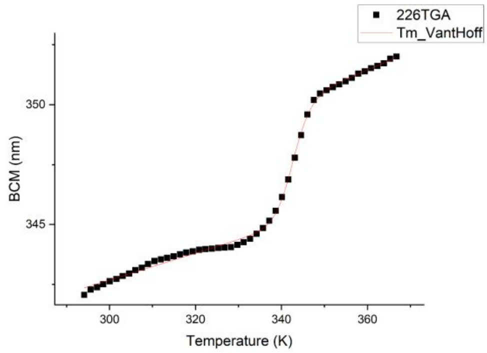
226TGA
Tm_VantHoff
BCM (nm)
350
345
300
320
340
360
Temperature (K)

**T**

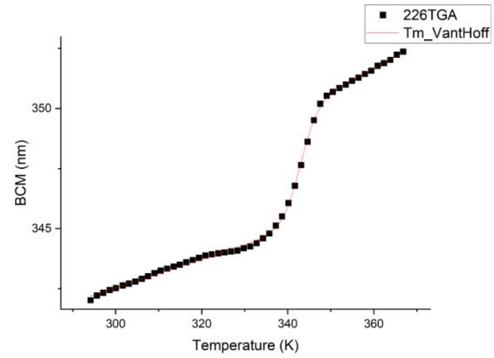
226TGA
Tm_VantHoff
BCM (nm)
350
345
300
320
340
360
Temperature (K)

**U**

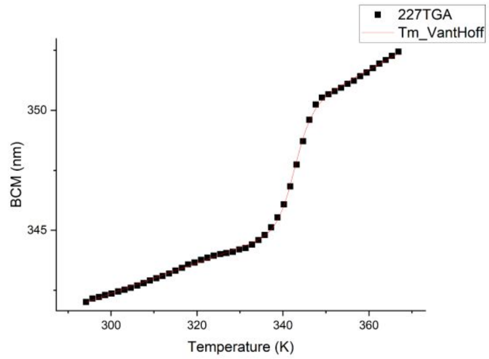
227TGA
Tm_VantHoff
BCM (nm)
350
345
300
320
340
360
Temperature (K)

**V**

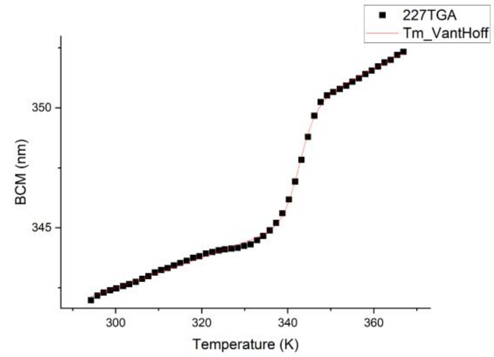
227TGA
Tm_VantHoff
BCM (nm)
350
345
300
320
340
360
Temperature (K)

**W**

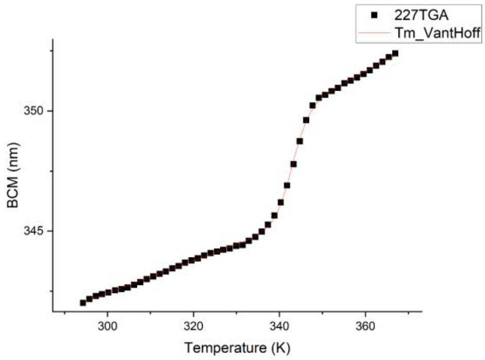
227TGA
Tm_VantHoff
BCM (nm)
350
345
300
320
340
360
Temperature (K)

**X**

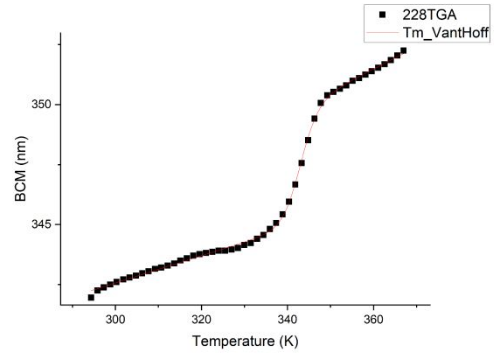
228TGA
Tm_VantHoff
BCM (nm)
350
345
300
320
340
360
Temperature (K)

**Y**

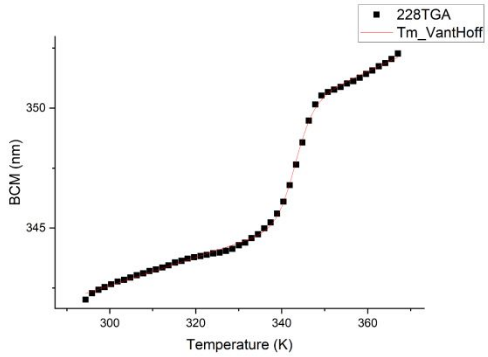
228TGA
Tm_VantHoff
BCM (nm)
350
345
300
320
340
360
Temperature (K)

**Z**

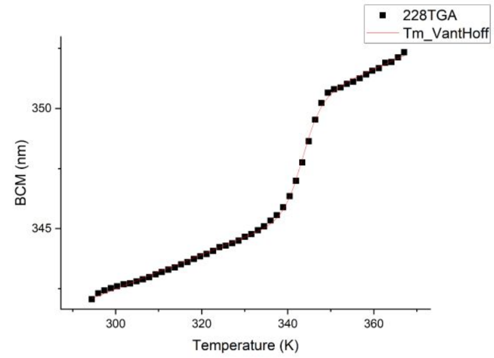
228TGA
Tm_VantHoff
BCM (nm)
350
345
300
320
340
360
Temperature (K)

**AA**

SI_Figure 2. Two-state fit to the Fab A33 thermal melting transitions in their intrinsic fluorescence barycentric mean emission wavelengths for A) WT replica 1, B) WT replica 2, C) WT replica 3, D) 221TGA replica 1, E), 221TGA replica 2, F) 221TGA replica 3, G) 222TGA replica 1, H) 222TGA replica 2, I) 222TGA replica 3, J) 223TGA replica 1, K), 223TGA replica 2, L) 223TGA replica 3, M) 224TGA replica 1, N), 224TGA replica 2, O) 224TGA replica 3, P) 225TGA replica 1, Q), 225TGA replica 2, R) 225TGA replica 3, S) 226TGA replica 1, T), 226TGA replica 2, U) 226TGA replica 3, V) 227TGA replica 1, W), 227TGA replica 2, X) 227TGA replica 3, Y) 228TGA replica 1, Z), 228TGA replica 2, AA) 228TGA replica 3.

*SI Table 4*. Statistics from two-state fits to the Fab A33 thermal melting transitions.

| **Variant replica** | $T_m$ | **SEM** | **Reduced Chi²** | **Residual Sum of squares** | $R^2$ |
|---|---|---|---|---|---|
| WT 1 | 69.8 | 0.30 | 0.02975 | 1.33895 | 0.99995 |
| WT 2 | 69.7 | 0.29 | 0.02923 | 1.31533 | 0.99995 |
| WT 3 | 69.7 | 0.29 | 0.02859 | 1.28645 | 0.99995 |
| 221TGA 1 | 66.9 | 0.44 | 0.01584 | 0.71261 | 0.99997 |
| 221TGA 2 | 66.9 | 0.60 | 0.02267 | 1.02033 | 0.99996 |
| 221TGA 3 | 66.5 | 0.54 | 0.02259 | 1.01647 | 0.99996 |
| 222TGA 1 | 67.6 | 0.28 | 0.02787 | 1.25418 | 0.99995 |
| 222TGA 2 | 67.4 | 0.39 | 0.05452 | 2.45352 | 0.9999 |
| 222TGA 3 | 67.8 | 0.33 | 0.04157 | 1.87082 | 0.99992 |
| 223TGA 1 | 70.1 | 0.18 | 0.0128 | 0.57621 | 0.99998 |
| 223TGA 2 | 70.0 | 0.22 | 0.01992 | 0.89655 | 0.99996 |
| 223TGA 3 | 70.5 | 0.13 | 0.00718 | 0.32305 | 0.99999 |
| 224TGA 1 | 70.4 | 0.20 | 0.01252 | 0.56337 | 0.99998 |
| 224TGA 2 | 70.7 | 0.13 | 0.00701 | 0.31566 | 0.99999 |
| 224TGA 3 | 70.6 | 0.10 | 0.00429 | 0.19299 | 0.99999 |
| 225TGA 1 | 69.6 | 0.18 | 0.01163 | 0.52357 | 0.99998 |
| 225TGA 2 | 69.7 | 0.14 | 0.0074 | 0.32546 | 0.99999 |
| 225TGA 3 | 69.9 | 0.11 | 0.00481 | 0.21148 | 0.99999 |
| 226TGA 1 | 69.4 | 0.19 | 0.01376 | 0.60537 | 0.99997 |
| 226TGA 2 | 69.5 | 0.19 | 0.01486 | 0.65363 | 0.99997 |
| 226TGA 3 | 69.7 | 0.13 | 0.00676 | 0.29745 | 0.99999 |
| 227TGA 1 | 69.5 | 0.11 | 0.00496 | 0.21834 | 0.99999 |
| 227TGA 2 | 69.5 | 0.13 | 0.00697 | 0.3067 | 0.99999 |
| 227TGA 3 | 69.8 | 0.10 | 0.00415 | 0.1824 | 0.99999 |
| 228TGA 1 | 69.8 | 0.15 | 0.00844 | 0.37135 | 0.99998 |
| 228TGA 2 | 70.0 | 0.16 | 0.00908 | 0.3994 | 0.99998 |
| 228TGA 3 | 70.4 | 0.15 | 0.0071 | 0.31262 | 0.99999 |

## 3.2 3-state fit for 221TGA and 222TGA

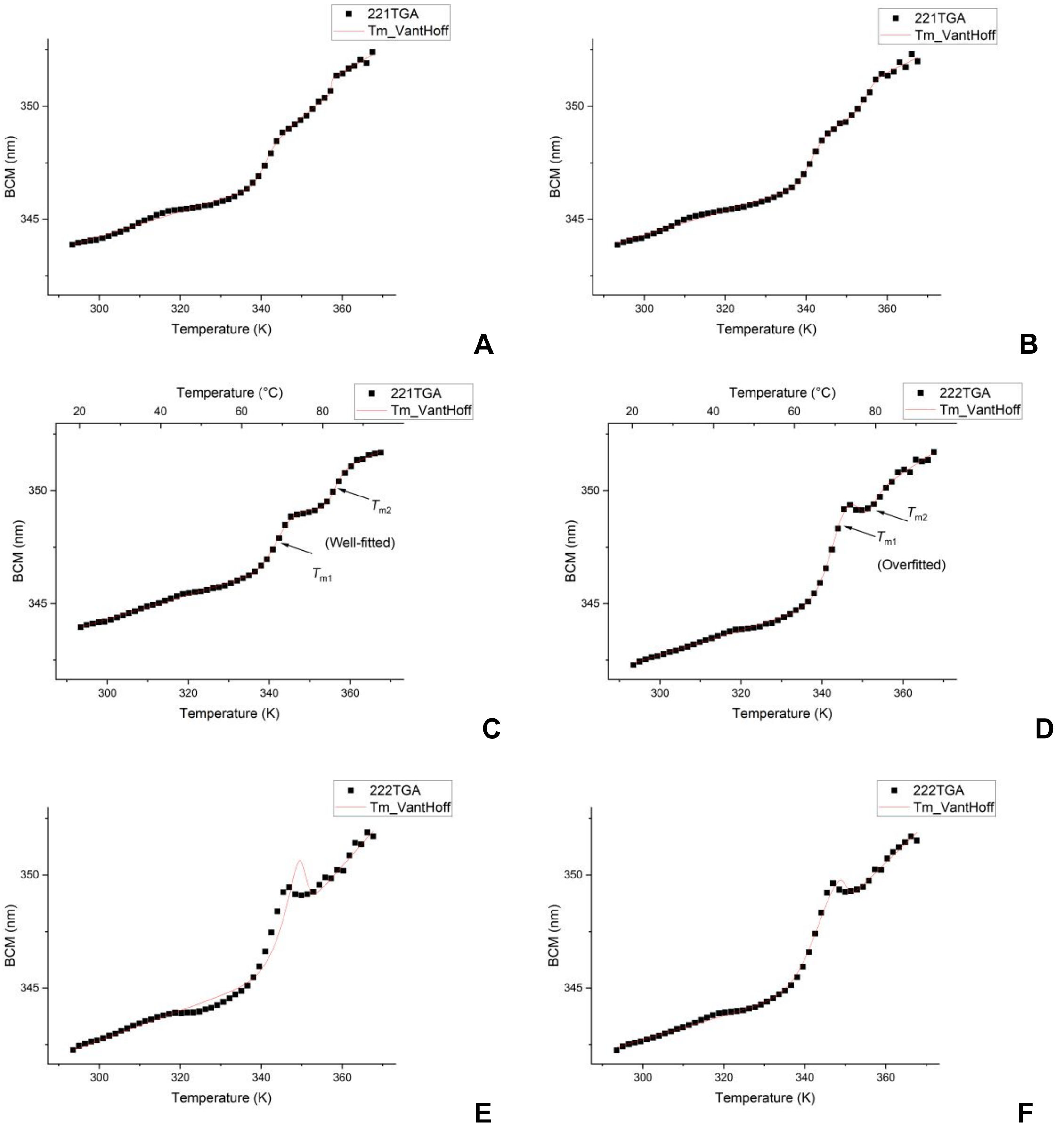


SI_Figure 3. Three-state fits to the Fab A33 thermal melting transitions in their intrinsic fluorescence barycentric mean emission wavelengths for A) 221TGA replica 1, B), 221TGA replica 2 C) 221TGA replica 3, D) 222TGA replica 1, E) 222TGA replica 2, F) 222TGA replica 3.

*SI Table 5*. Statistics from three-state fits to the Fab A33 thermal melting transitions. * SEM values of 0 indicate overfitting.

| Variant replica | $T_m$1 | SEM | $T_m$2 | SEM | Reduced Chi$^2$ | Residual Sum of squares | $R^2$ |
|---|---|---|---|---|---|---|---|
| 221TGA 1 | 67.3 | 0.4 | 84.1 | 16.3 | 0.00988 | 0.40517 | 0.99998 |
| 221TGA 2 | 67.9 | 0 * | 81.6 | 0 * | 0.01108 | 0.45438 | 0.99998 |
| 221TGA 3 | 69.0 | 0 * | 82.8 | 0 * | 0.00349 | 0.14319 | 0.99999 |
| 222TGA 1 | 71.0 | 0 * | 78.3 | 0 * | 0.00794 | 0.32569 | 0.99999 |
| 222TGA 2 | 75.5 | 18.3 | 76.9 | 4.2 | 0.18524 | 7.59501 | 0.99969 |
| 222TGA 3 | 70.5 | 5.8 | 76.9 | 1.1 | 0.02475 | 1.01467 | 0.99996 |

# 4 SEC-HPLC retention time

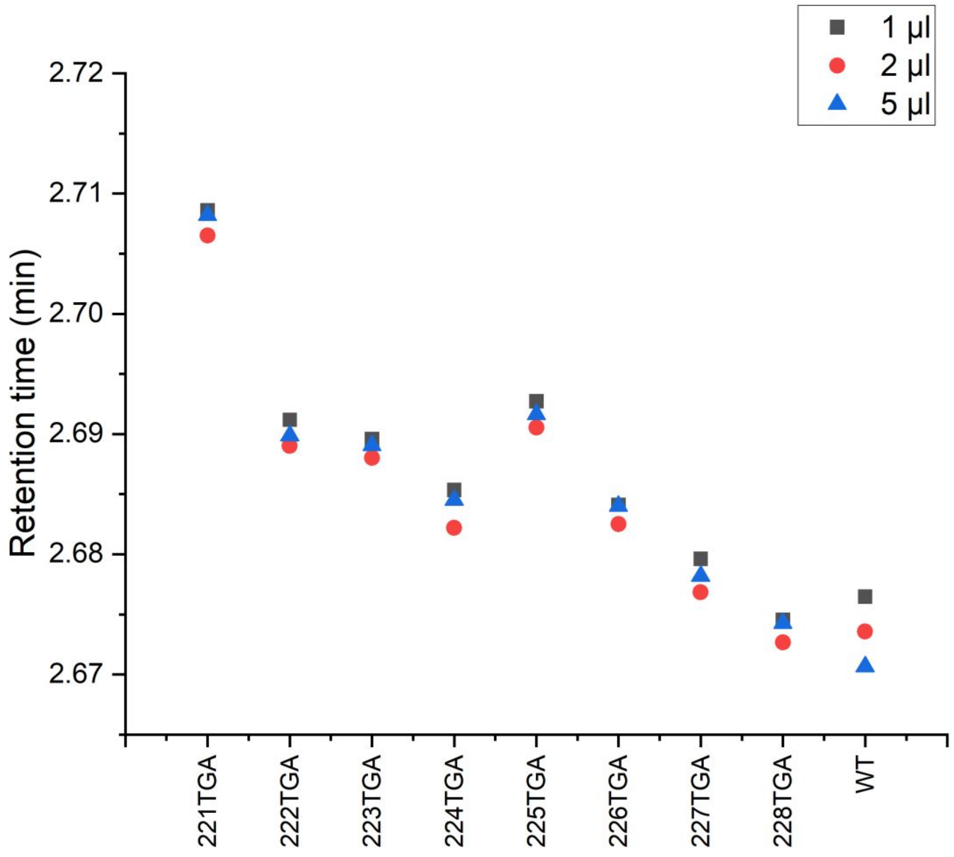


SI_Figure 4. SEC-HPLC Retention Times for Calibration Samples Across Different Variants

Retention times measured for various variants labelled 221TGA through 228TGA and WT (wild type), analysed using SEC-HPLC. Calibration samples were prepared at a concentration of 2 mg/ml and injected in volumes of 1 µL (black squares), 2 µL (red circles), and 5 µL (blue triangles). This analysis was performed to indirectly compare the protein sizes of each variant under identical conditions.
